# Mapping the Influence of Light Intensity on the Transgenerational Genetic Architecture of *Arabidopsis thaliana*

**DOI:** 10.3390/cimb46080482

**Published:** 2024-07-29

**Authors:** Jie Mei, Jincan Che, Yunzhu Shi, Yudian Fang, Rongling Wu, Xuli Zhu

**Affiliations:** 1 Center for Computational Biology, College of Biological Sciences and Technology, Beijing Forestry University, Beijing 100083, China; jiemei103@bjfu.edu.cn (J.M.); chejincan@bjfu.edu.cn (J.C.); yunzhushi202@bjfu.edu.cn (Y.S.); yudianfang@bjfu.edu.cn (Y.F.); rwu@bjfu.edu.cn (R.W.); 2 National Engineering Research Center of Tree Breeding and Ecological Restoration, Beijing Forestry University, Beijing 100083, China

**Keywords:** transgenerational, functional mapping, quantitative trait loci (QTL), light intensity

## Abstract

Light is a crucial environmental factor that influences the phenotypic development of plants. Despite extensive studies on the physiological, biochemical, and molecular mechanisms of the impact of light on phenotypes, genetic investigations regarding light-induced transgenerational plasticity in *Arabidopsis thaliana* remain incomplete. In this study, we used *thaliana* as the material, then gathered phenotypic data regarding leaf number and plant height under high- and low-light conditions from two generations. In addition to the developed genotype data, a functional mapping model was used to locate a series of significant single-nucleotide polymorphisms (SNPs). Under low-light conditions, a noticeable adaptive change in the phenotype of leaf number in the second generation suggests the presence of transgenerational genetic effects in *thaliana* under environmental stress. Under different lighting treatments, 33 and 13 significant genes associated with transgenerational inheritance were identified, respectively. These genes are largely involved in signal transduction, technical hormone pathways, light responses, and the regulation of organ development. Notably, genes identified under high-light conditions more significantly influence plant development, whereas those identified under low-light conditions focus more on responding to external environmental stimuli.

## 1. Introduction

Because of sessile growth, plants change their development and physiological characteristics under various environmental stresses to better adapt to the current environment [1]. This plasticity can be transmitted across generations, wherein the phenotype of an offspring is influenced by the environmental conditions experienced by the maternal plant, a phenomenon referred to as transgenerational plasticity [2]. Transgenerational plasticity is mainly achieved through epigenetic mechanisms, including but not limited to DNA methylation, histone modification, and noncoding-RNA-mediated mechanisms [3]. In the expression of some abnormal phenotypes, this influence is even stronger than that of DNA inheritance [4]. This inheritance mode extends beyond conventional Mendelian genetics and encompasses the effects of environmental factors, lifestyles, and other nongenetic elements on offspring phenotypes.

*Arabidopsis thaliana*, a model plant in the field of botany, is highly regarded for its unique advantages. Researchers have extensively explored the transgenerational inheritance of phenotypes in this species. When recombinant *thaliana* inbred lines were cultivated under mild heat conditions (30 °C), the seed yield of the third generation surpassed that of the first generation by more than fivefold [5]. Under high-temperature stress, the flowering time and plant structure of *thaliana* progenies exhibit substantial alterations, enhancing their adaptation to high-temperature environments [6]. The fruit-quality fraction of the progeny of the ancestral plants in a low-nutrient environment was significantly higher than that of the progeny of the ancestral plants in a suitable nutrient environment [7]. These findings collectively suggest that *thaliana* retains a non-DNA-inherited memory of ancestral environmental conditions, subsequently enhancing offspring adaptability. This memory appears to be transmitted through epigenetic inheritance mechanisms [8]. Intergenerational plasticity plays a pivotal role in enhancing offspring adaptability within the same environmental context, thereby notably bolstering plant survival capabilities under continuous environmental challenges [9,10].

Light exerts a profound influence on plant phenotypic development through signal sensing, signal transmission, and hormone regulation [11,12]; these responses to light conditions can also be transmitted to subsequent generations. For instance, in *Wedelia trilobata*, offspring subjected to low-light treatment displayed significantly increased leaf area, potential maximum net photosynthetic rates, and biomass accumulation, compared with those under high-light conditions [13]. The offspring of *Vicia faba* L. exposed to short ultraviolet light for a long time established a protective mechanism through the accumulation of flavonoids in the early stage [14]. Projections for population growth in wild plants revealed that those adapted to their current light environment through maternal effects exhibit 3–4 times higher adaptability than other plants [2]. In *thaliana*, a plethora of light-responsive signal receptors, growth-influencing biohormones, and enzymes and proteins that modulate biohormones have been identified [15,16,17]. However, genetic studies investigating the effects of light on transgenerational plasticity in *thaliana* remain limited. Key areas such as the genes that play pivotal roles in transgenerational plasticity and those that exert indirect effects on this phenomenon warrant further exploration.

In this study, we employed a statistical model to quantitatively assess the genetic influences underlying transgenerational plasticity within the context of functional mapping. Using *thaliana* as our study material, we aimed to identify the transgenerational plasticity gene by analyzing *thaliana* genotypes and dynamic growth phenotypes across two generations subjected to high- and low-light treatments to elucidate the genetic mechanisms underlying the transgenerational phenotypic plasticity of *thaliana* in response to varying light conditions.

## 2. Materials and Methods

### 2.1. Experimental Materials

In this study, hybrid progeny derived from *thaliana Ler* (*Landsberg erecta*) and *Shr* (*Shahdara*) were used as parental materials. The primary phenotypic characteristics of *Ler* include round leaves, short petioles, short pedicels, and a plant height ranging from approximately 10 to 25 cm. *Shr* is characterized by narrow leaves and a plant height of approximately 30 cm. Through ten consecutive generations of selfing, a total of 84 recombined inbred lines (RILs) were generated, resulting in a highly pure population. This meticulously bred population served as an ideal resource for genetic mapping and QTLs localization.

**Figure 1:**
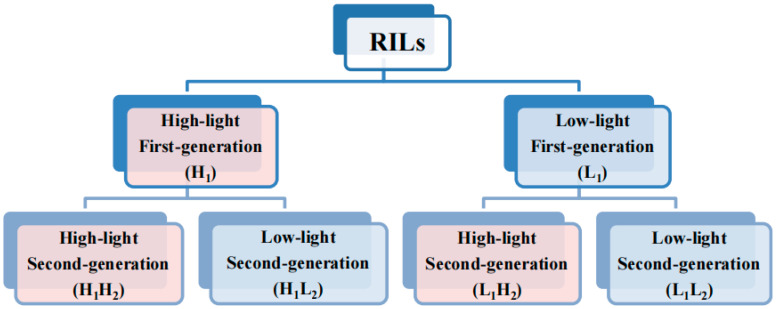
Simplified experimental workflow diagram. Light red and blue represent the high- and low-light treatments, respectively. The second and third rows represent the first-and second-generation populations of *Arabidopsis thaliana*, respectively. The abbreviations in parentheses denote the population names, with the specific abbreviation rule as follows: H and L represent high- and low-light conditions, respectively; 1 and 2 denote first- and second- generation populations, respectively. For instance, ‘H_1_’ denotes a first-generation population subjected to high-light treatment, ‘H_1_H_2_’ designates a second-generation population derived from a first-generation population exposed to high-light treatment, with the second-generation population similarly grown under high-light conditions. Similarly, ‘H_1_L_2_’ signifies a second-generation population originating from a first-generation population exposed to high-light treatment, but with the second-generation population cultivated under low-light conditions.

This study was initiated by sowing seeds from the RIL population consisting of 84 distinct lines. These seeds were cultivated to expand the population and subsequently divided into two groups, with each line having a total of 20 replicates. *Thaliana* seedlings were nurtured under controlled conditions, maintaining a constant temperature of 22 °C, 80% relative humidity, 16 h of daily light exposure, and a nutrient-rich soil mixture comprising charcoal ash, limestone, and vermiculite in a 1:1:1 ratio. The high-light group received daily illumination of 7633 lux, whereas the low-light group was exposed to 4852 lux. Subsequently, first-generation *thaliana* populations under two different light treatments were obtained. Then, the seeds from both first-generation populations were propagated and subjected to high- and low-light treatments as described above, resulting in four second-generation *thaliana* populations. In summary, the entire process yielded six groups of populations under different treatments, as illustrated in the simplified flowchart in Figure 1.

### 2.2. Phenotypic Data Acquisition

Phenotypic data, including measurements of plant height and total leaf count, were meticulously recorded at regular intervals starting from one week after planting and, subsequently, every seven days. The phenotypic values for leaf number included both rosette and cauline leaves, whereas the plant height phenotype was described using the primary inflorescence height. Comprehensive phenotypic data were amassed, capturing the leaf number over seven measurement intervals and the plant height over eight measurement intervals. 

### 2.3. Genotypic Data Acquisition

After gathering the phenotypic data from *thaliana*, we extracted the genomic DNA from the entire recombined inbred line (RIL) population. Whole-genome resequencing was conducted using the Illumina HiSeq2000 platform at Shenzhen Hengchuang Gene Technology Co., Ltd., Shenzhen, China. The *thaliana* reference genome data were downloaded from https://ftp.ncbi.nlm.nih.gov/genomes/all/GCF/000/001/735/GCF_000001735.4_TAIR10.1/GCF_000001735.4_TAIR10.1_genomic.fna.gz, accessed on 1 October 2022. To ensure the quality of the raw data, a BWA bioinformatic analysis software (bwa-0.7.17) was employed for alignment. Subsequently, SAMTOOLS (samtools-1.11) was used to detect single-nucleotide polymorphism (SNP) variants at the population level. The SNPs were filtered using VCFtools (vcftools-0.1.13) with the following criteria: a quality value of comparison ≥ 20, a quality value of variant detection ≥ 50, a sequencing depth > 12, a *p*-value of the Hardy–Weinberg equilibrium test > 5%, a minimum allele frequency > 5%, and deletion and heterozygosity rates < 10%. This process resulted in the identification of 164,029 high-quality SNPs.

### 2.4. Phenotypic Data Processing and Analysis

After phenotypic data were gathered from 20 replicates within each lineage and group at the designated measurement time point, the mean phenotypic value for each lineage was computed based on these 20 replicates. For the high-light first-generation population, any missing measurement data were excluded, resulting in a final set of phenotypic values for 79 genotypes. In accordance with our experimental design, comparisons among different phenotypic combinations were employed to characterize the plasticity relationships across generations. The specific plasticity relationships were categorized and abbreviated as described in Table 1.

To conduct a variation statistical analysis of the phenotype data, an R package MVN version 5.9 was used to analyze the phenotype data for each group, obtaining statistical metrics including mean, standard deviation (SD), range, coefficient of variation (CV), and kurtosis. Additionally, an R package reshape2 version 1.4.4 was employed to organize the data, which was followed by visualizing the overall variation in the phenotype data in the form of box plots using an R package ggplot2 version 3.5.1. To further compare the phenotypic growth over time under the different light treatments, histograms of the mean values for each group of phenotype data were plotted. The *t*-test function in R version 4.1.0 was employed to detect the existence of any significant differences in the mean values of the phenotypes under the different light treatments at the different measurement time points. Microsoft Excel version 2108 was used to integrate the mean values, standard deviations, and significant *p*-values into a wide data format suitable for plotting. The R version 4.1.0 packages, ggplot2 version 3.5.1 and ggpubr version 0.6.0, were used for visualizing the histograms.

### 2.5. Functional Mapping

To localize QTLs, a statistical framework–functional mapping [18] was proposed. The functional mapping model includes fitting biologically meaningful functions, parameter estimation, hypothesis testing, and filtering to obtain the target QTL. A logistic growth model was initially used to study the growth of the biological populations, which is practically valuable and is now widely used in economics, biology, medicine, and other fields [19]. In this study, the plant height and leaf number phenotypes were be fitted by the following equation:

(1) $$g\left(t\right)=\frac{a}{1+be^{-rt}}$$

where g is the asymptotic or limit value of g when  t→∞, a/(1+b)  is the initial value of *g* when *t* = 0, and *r* is the relative rate of growth.

Let $y_{i}$ be the phenotypic data measured at time point $\;T(t_{1},t_{2},\ldots ,t_{T})$ for line number i. The sample size of the mapping population is *n*, and *p* marker loci are obtained after gene sequencing. For a particular SNP locus, a genotyping $j\left(j=1,2,\ldots ,J\right)$ exists; the phenotypic data of all samples under genotypic component j at time point *T* obey a multivariate normal distribution:

(2) $$f_{i}\left(y_{i}\right)=\frac{1}{{\left(2\pi \right)}^{T/2}{\left|\Sigma \right|}^{1/2}}exp\left(-\frac{1}{2}{\left(y_{i}-u_{j}\right)}^{T}\Sigma^{-1}\left(y_{i}-u_{j}\right)\right)$$

where the fitted mean $u_{j}$ was obtained from Equation (1) ${(u}_{j}=\left(g\left(t_{1}\right),g\left(t_{2}\right),\ldots ,g\left(t_{T}\right)\right)$, and the *Σ* covariance matrix was modeled using one-order structured ante-dependence model (SAD1) [20]:

(3) $$\Sigma =\sigma^{2}\left[\begin{array}{cc} \begin{array}{cc} 1 & \rho \\ \rho & 1 \end{array} & \begin{array}{cc} \cdots & \rho^{T-1}\\ \cdots & \rho^{T-2} \end{array}\\ \begin{array}{cc} \cdots & \cdots \\ \rho^{T-1} & \rho^{T-2} \end{array} & \begin{array}{cc} \ddots & \cdots \\ \cdots & 1 \end{array} \end{array}\right]$$

For all genotypes j of a given SNP, by integrating the above growth curves, covariance matrices, and multivariate normal distribution functions, the likelihood function is constructed as follows:

(4) $$L\left(Y\right)=\prod_{j=1}^{J}\prod_{i=1}^{n_{j}}f\left(y_{i};u_{j};\Sigma \right)$$

When the display solution is not directly available, some optimization algorithms such as Simplex and BFGS [21,22] can be used to estimate the unknown parameters (a, b, r, σ, ρ) in the likelihood function.

To determine whether a QTL significantly affects phenotypic variation, the judgment criterion is set as whether the means of different components under different genotypes are significantly different. This hypothesis can be tested as follows:

(5) $$\begin{array}{r} H_{0}:u_{j}=u\;\;\;\;\;\;\;\;\;\;\;\;\;\\ H_{1}:not\;all\;equations\;apply\;to\;H_{o} \end{array}$$

After computing the likelihood values under each hypothesis, the logarithm of the likelihood ratio for each locus is obtained using the following formula:

(6) $$LR=-2\log\left[\frac{L_{0}(Y)}{L_{1}(Y)}\right]$$

Considering the statistical uncertainty of the probability distribution of likelihood ratio *(LR)*, in this hypothesis testing, 1000 permutation tests were used to determine the threshold value and filtered to obtain the significant QTL. The R function, pchisq, was employed to convert *LR* values into *p*-values, and the R package, qqman version 0.1.9, was used for visualizing the quantile–quantile plots. All the above calculations were performed using R version 4.1.0 [23].

### 2.6. Construction of Genetic Effect Networks

The Lotka–Volterra (LV) model framework was first proposed by Alfred Lotka and Vito Volterra to describe the dynamics of predator–prey interactions in biological systems. As research progressed, the LV equations have been shown to be a powerful tool for analyzing interactions within various biological systems [24]. Based on the LV model framework, following the approach of Yang et al. [25], the independent genetic effects of different SNP loci are considered as nodes and the dependent genetic effects as edges to construct a genetic effect network diagram of significant loci. All calculations were performed in R version 4.1.0, and the network diagram was visualized using Cytoscape version 3.7.1 [26].

### 2.7. Functional Annotation

To gather detailed information on each identified significant QTL, we aligned the significant SNPs with the genome annotation file. This alignment allowed us to compile a comprehensive list of the significant gene IDs associated with these loci. The gene descriptions for each of these significant gene IDs are accessible through Genome Data Viewer on the National Center for Biotechnology Information (NCBI)’s website (https://www.ncbi.nlm.nih.gov/, accessed on 12 October 2023). To further understand the biological processes involved in the significant loci, the list of gene IDs was input to R software and compared with the org.at.tair.db gene-annotation package to convert the IDs. Finally, we performed a GO analysis using the R package, clusterProfiler version 4.12.1, and selected the gene entries related to the target traits and with a high number of enriched genes to draw the GO annotation maps.

### 2.8. Genetic Effects Analysis of Significant Genes

By leveraging the powerful statistical capabilities of the functional mapping model, the genetic effects of different genotypes of significant genes can be analyzed. The specific procedure involved likelihood calculations based on Equation (4) to obtain the estimated parameters for the different genotypes of each SNP. Using the *Arabidopsis* genotype file, where each SNP exists in AA and aa genotypes, respectively, the estimated parameters for different genotypes are substituted into Equation (1) to fit the overall growth curve of the corresponding gene with different genotypes.

## 3. Results

### 3.1. Analysis of Phenotypic Variation under Different Light Conditions

The statistical data for each set of phenotypic data regarding leaf number and plant height traits are summarized in Tables S1 and S2, respectively. The statistical distributions are visualized in Figures S1–S3. At each time point, the standard deviation accounted for 7% to 18% of the mean, with this proportion diminishing as the plants continued to grow. The coefficient of variation for leaf phenotypic data consistently remained below 0.2, indicating relatively concentrated data. In contrast, the coefficient of variation for the plant height phenotypic data ranged from 0.08 to 1.05. This disparity underscored substantial variations in the growth rates of individual *thaliana* plants over time, particularly notable among the first-generation phenotypic data. Most time points exhibited kurtosis coefficients below 0, indicative of less-concentrated central data. However, the kurtosis coefficient at the third measurement time point for plant height was significantly higher than those at the other measurement time points. This divergence was attributed to this particular time point, marking the juncture at which the growth rate of *thaliana* accelerated. Notably, the coefficient of variation highlighted substantial growth rate differences among individual plants at this juncture, resulting in extreme deviations from the mean and consequently elevating the kurtosis coefficient.

**Figure 2:**
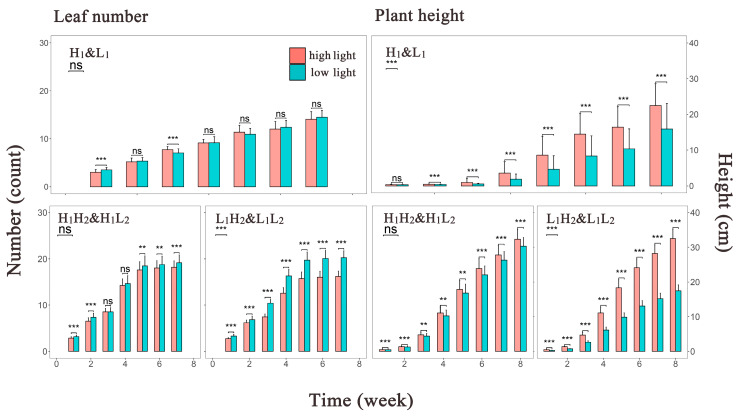
Histograms depicting the mean phenotype values of *Arabidopsis thaliana* over time. The left panel shows the mean leaf number over time, whereas the right panel displays the mean plant height over time. The *x* axis represents the measurement time points, and the *y* axis represents the phenotype values. Red and green bars represent the high- and low-light treatments, respectively. The central line within each bar is the error bar, and above each bar, the central line indicates the significance level of the mean differences between the two treatments at each time point. ns, *p* > 0.05; **, *p* ≤ 0.01; ***, *p* ≤ 0.001. In the top left corner of this Figure, the significance level and abbreviations for the groups are provided, with abbreviations detailed in Section 2.1.

To depict the overall trend in phenotype growth over time under different light environments, bar graphs displaying the mean values for all phenotype groups were constructed (Figure 2). We found almost no difference in the leaf number generated by the first-generation populations under different light environments. Similarly, the difference in leaf number among the second-generation populations under high-light treatment was not pronounced until the late growth stage, where slightly more leaves were observed in the low-light environment than in the high-light environment. The second-generation population under low-light treatment from the first generation exhibited the largest disparity in leaf number among all phenotype groups, with the leaf count in the L_1_L_2_ group being the highest. In contrast, plant height exhibited significant differences among the first-generation populations, with little development observed in the early stages but more pronounced growth observed under high-light conditions from the mid-growth stage onward. This phenotypic variation induced by different environments was more evident among the second-generation populations under the low-light treatment. Conversely, although the plant height of H_1_H_2_ group gradually surpassed that of the H_1_L_2_ group over time, the disparity compared with the other two groups was not substantial. Notably, plant development occurred earlier in the second-generation than in the first-generation populations.

### 3.2. Significant SNPs Located Via Functional Mapping

**Figure 3:**
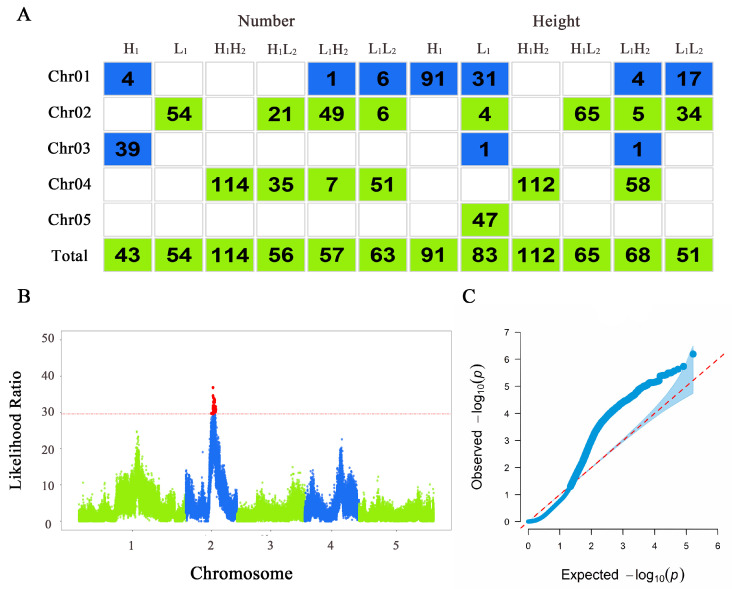
Results of functional mapping. (**A**) Distribution of significant loci across chromosomes in various populations of *Arabidopsis thaliana*. Each column represents the various populations under different treatments. The first five rows indicate the number of significant loci mapped on each chromosome, and the last row shows the total significant loci mapped for each population. (**B**) Manhattan plot of plant height trait of *thaliana* in the H_1_L_2_ population. The *x* axis represents the chromosome position where the SNP locus is located, and the *y* axis represents the likelihood ratio statistic of the SNP locus; the red dashed line represents the threshold obtained from the 1000 permutation assay; and the red loci on top of it represent the SNP loci significantly correlated with the phenotype. (**C**) Quantile–quantile plot of plant height of *thaliana* in the H_1_L_2_ population. The *x* axis represents the expected distribution, and the *y* axis represents the observed distribution.

The QTLs that play a dominant role in determining the leaf number and plant height of *thaliana* in response to different light conditions were localized via functional mapping, and the results were plotted in Figure 3. The Manhattan plots and quantile–quantile plots were shown in Figures S4 and S5. H_1_ localized 43 significant SNPs; L_1_ localized 54 significant SNPs; H_1_H_2_ localized 114 significant SNPs; H_1_L_2_ localized 56 significant SNPs; L_1_H_2_ localized 57 significant SNPs; and L_1_L_2_ localized 63 significant SNPs. For plant height, H_1_ localized 91 significant SNPs; L_1_ localized 83 significant SNPs; H_1_H_2_ localized 112 significant SNPs; H_1_L_2_ localized 65 significant SNPs; L_1_H_2_ localized 68 significant SNPs; and L_1_L_2_ localized 51 significant SNPs. Overall, the positional distribution of the significant loci on chromosomes varied, with chromosomes 2 and 4 containing a larger number of significant SNPs.

After comparing the localized significant SNPs with the genome annotation files, we identified the final annotated significant gene IDs associated with leaf number: 32 in the H_1_ group, 38 in the L_1_ group, 48 in the H_1_H_2_ group, 40 in the H_1_L_2_ group, 38 in the L_1_H_2_ group, and 35 in the L_1_L_2_ group. Subsequently, we conducted gene description queries for each of these significant gene IDs through the NCBI online website and organized the results into appendices (Tables S3–S14).

**Figure 4:**
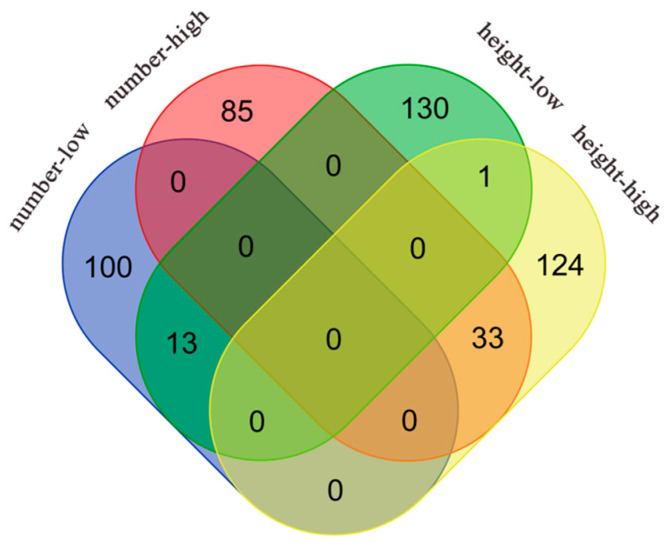
Venn diagram showing significant genes associated with leaf number and plant height traits in *Arabidopsis* under different light treatments. The red ellipse represents significant genes associated with leaf number trait under high-light treatment, the blue ellipse represents significant genes associated with leaf number trait under low-light treatment, the yellow ellipse represents significant genes associated with plant height trait under high-light treatment, and the green ellipse represents significant genes associated with plant height trait under low-light treatment.

The significant gene IDs obtained from the above analysis for leaf number and plant height in the high- and low-light groups were input to a website (https://bioinformatics.psb.ugent.be/webtools/Venn/, accessed on 25 October 2023) to draw a Venn diagram (Figure 4) to search for significant genes associated with both leaf number and plant height under the same environment and to find the significant genes associated with both leaf number and plant height traits through the NCBI online website using Genome Data Viewer to find their specific gene descriptions, which are plotted in Table 2 and Table 3.

A total of 33 and 13 significant genes were duplicated in high- and low-light conditions, respectively, for *thaliana* leaf number and plant height. All of the duplicated genes under high light are distributed on chromosome 4, whereas most of those duplicated under low light are located on chromosome 2, which corresponds to the results shown in the Manhattan plot. Most of the duplicated genes in both environments could be categorized according to functional annotation into four groups: developmental regulation, stress response, transcriptional regulation, and anabolic correlation. For example, among the duplicated genes under high light, *AT4G30840*, *AT4G34220*, *AT4G35390,* and *AT4G35840* are related to developmental regulation, and *AT2G14210*, *AT4G35920*, *AT4G36150,* and *AT4G36140* are related to stress response. *AT4G35890*, *AT4G36590,* and *AT4G36650* are related to transcriptional regulation, and *AT4G35650*, *AT4G35640,* and *AT4G36090* are related to anabolism. The genes duplicated under low light also included *AT1G16440* and *AT2G27520*, which are related to developmental regulation; *AT1G16670*, *AT2G27580,* and *AT2G28060,* which are related to stress response; *AT2G27700* and *AT2G27880*, which are related to transcriptional regulation; and *AT2G18640*, *AT2G27570*, and *AT2G27650,* which are related to anabolism. Simultaneously, when exposed to ample light resources, plants seem to prioritize individual development, entailing the regulation of various hormonal pathways. Conversely, in conditions of limited light availability, plants tend to be more responsive to external environmental stimuli.

### 3.3. Genetic Effect Network Analysis

The genetic effect network diagram (Figure S6) provides a new perspective on our understanding of gene function. For leaf number traits, there are 4 hub genes in the H_1_ generation, 6 in the L_1_ generation, 2 in the H_1_H_2_ generation, 3 in the H_1_L_2_ generation, 3 in the L_1_H_2_ generation, and 3 in the L_1_L_2_ generation. For plant height traits, there are 3 hub genes in the H_1_ generation, 6 in the L_1_ generation, 4 in the H_1_H_2_ generation, 3 in the H_1_L_2_ generation, 8 in the L_1_H_2_ generation, and 4 in the L_1_L_2_ generation. Most of these hub genes are associated with developmental regulation and signal transduction. For instance, in the second-generation population of leaf number traits, there are numerous hub genes encoding Transducin/WD40 repeat-like superfamily proteins. These proteins are particularly widespread in chromatin modification and transcription mechanisms, participating in various cellular and organismal processes, including cell division, cytokinesis, apoptosis, and light signal transduction [27]. In the context of plant height traits, there is a notable presence of hub genes encoding enzymes related to signal transduction. For instance, both the H_1_H_2_ and H_1_L_2_ generations exhibit hub genes encoding alpha/beta-Hydrolases superfamily proteins. The α/β-Hydrolases (ABH) superfamily represents a widely distributed and functionally adaptable protein folding structure. In plants, it serves as a foundational structure in various hormone pathways and ligand receptors, demonstrating robust functional versatility [28]. The interaction patterns among genes within each population’s network diagrams also exhibit complex and dynamic relationships.

**Figure 5:**
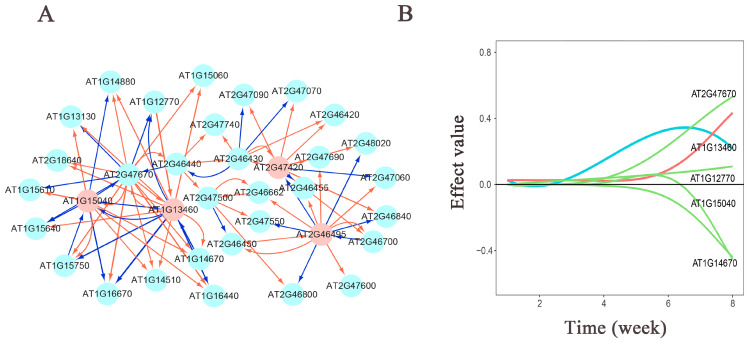
(**A**) Genetic network diagram of significant gene effects on plant height in the *Arabidopsis* L_1_L_2_ population. Pink nodes represent hub genes. Red arrows indicate promoting effects, while blue arrows indicate inhibitory effects. The thicker the arrow, the greater the influence; conversely, the thinner the arrow, the smaller the influence. (**B**) Genetic effect decomposition plot for *AT1G13460*. The horizontal axis represents the measurement time points, and the vertical axis represents the genetic effect values. Blue represents the actual effect curve, red represents the independent effect curve, and green represents the dependent effect curve.

In the population of the L_1_L_2_ group for plant height traits (Figure 5), we observed that the significant gene interactions exhibit more promotion than inhibition, with inhibitory effects predominantly exerted by hub genes on other connected genes. The hub genes *AT1G15040* and *AT1G13460* primarily exhibit inhibitory effects on other connected genes, and these two hub genes mutually inhibit each other. The hub gene *AT2G47420* is more prone to strong promoting effects, with inhibitory effects observed only on two connected genes, both of which are influenced by the promoting effect of the hub gene *AT2G46495*. Additionally, the hub gene *AT2G46495* directly inhibits the hub gene *AT2G47420*. From the effect decomposition diagram of *AT1G13460* (Figure 5A), it is evident that other genes have a greater influence on the *AT1G13460* gene, compared to its independent effect. The gene *AT2G47670* mainly promotes its expression, while the genes *AT1G15040* and *AT1G14670* inhibit its expression.

### 3.4. Gene Ontology (GO) Analysis

**Figure 6:**
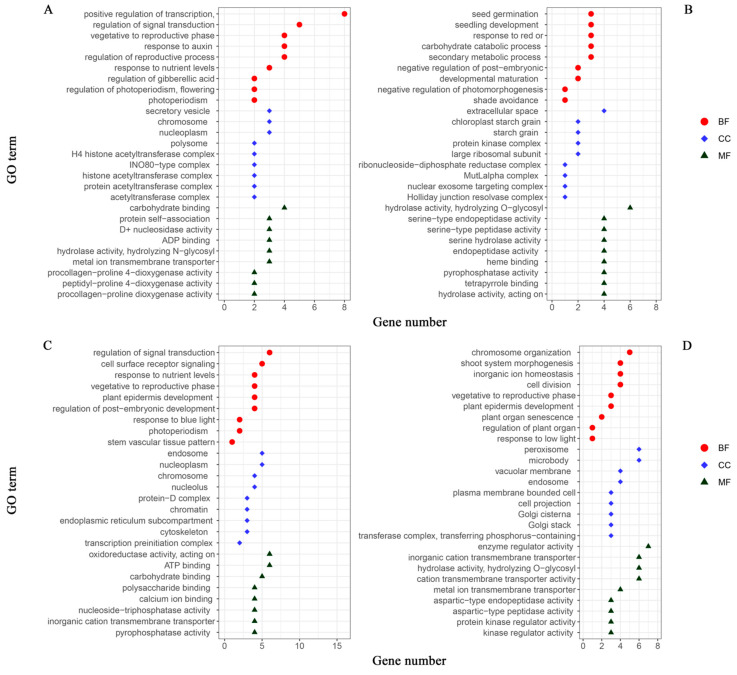
GO annotation maps of significant genes for growth traits in *Arabidopsis thaliana*: leaf number in (**A**) high-light and (**B**) low-light groups; plant height in (**C**) high-light and (**D**) low-light groups. The horizontal coordinate is the number of genes enriched to the GO entries, and the vertical coordinate is the content of the GO entries. Red circle represents biological processes (BP); blue diamond represents cellular components (CC); green triangle represents molecular functions (MF).

The phenotypic combinations of the two growth traits were stratified into high- and low-light environments to conduct a comprehensive analysis. The plant populations grown under high-light conditions (comprising H_1_, H_1_H_2_, and L_1_H_2_) and low-light conditions (consisting of L_1_, H_1_L_2_, and L_1_L_2_) were categorized as the high- and low-light groups, respectively. Subsequently, the significant gene IDs for each of the localized populations associated with leaf number and plant height were consolidated into the following groups: 118 and 113 significant gene IDs for leaf number in the high- and low-light groups, respectively; 158 and 144 significant gene IDs for plant height in the high- and low-light groups, respectively. To facilitate the analysis, we imported the list of gene IDs into RStudio2022.7.1.554 software and conducted ID conversion using the org.at.tair.db gene annotation package. Following this, we performed GO analysis using the R package, clusterProfiler. This analysis allowed us to select gene entries pertinent to the target traits that were enriched with a substantial number of genes, which we then used to construct GO annotation maps (Figure 6). The GO analysis, through which we identified the biological process (BP), cellular component (CC), and molecular function (MF), revealed the main biological functions and potential regulatory mechanisms of the genes that are significantly expressed under different light treatments, providing a foundation for further experimental design and theoretical research.

Key insights include the critical roles of genes in regulating transcription, chromosome organization, protein complexes, seed germination, and carbohydrate metabolism, indicating their importance in gene expression regulation, plant development, and energy metabolism. Additionally, the data highlight the genes involved in signal transduction and cellular responses to stimuli, underscoring their pivotal roles in cellular adaptation to environmental changes. The charts also emphasize the localization of gene products to specific cellular structures, such as the cell membrane and nucleus, reflecting their integral roles in maintaining cellular architecture and function. The presence of genes involved in both positive and negative regulation across these processes points to the complexity and precision of regulatory networks within biological systems. Furthermore, the distribution of gene numbers may reflect the foci of this study or the effects of specific experimental conditions, suggesting how certain treatments may influence gene expression in particular biological processes. The results of this comprehensive analysis provide foundational insights for further experimental design and theoretical research in this field.

### 3.5. Genetic Effects Analysis of Significant Genes across Different Populations

**Figure 7:**
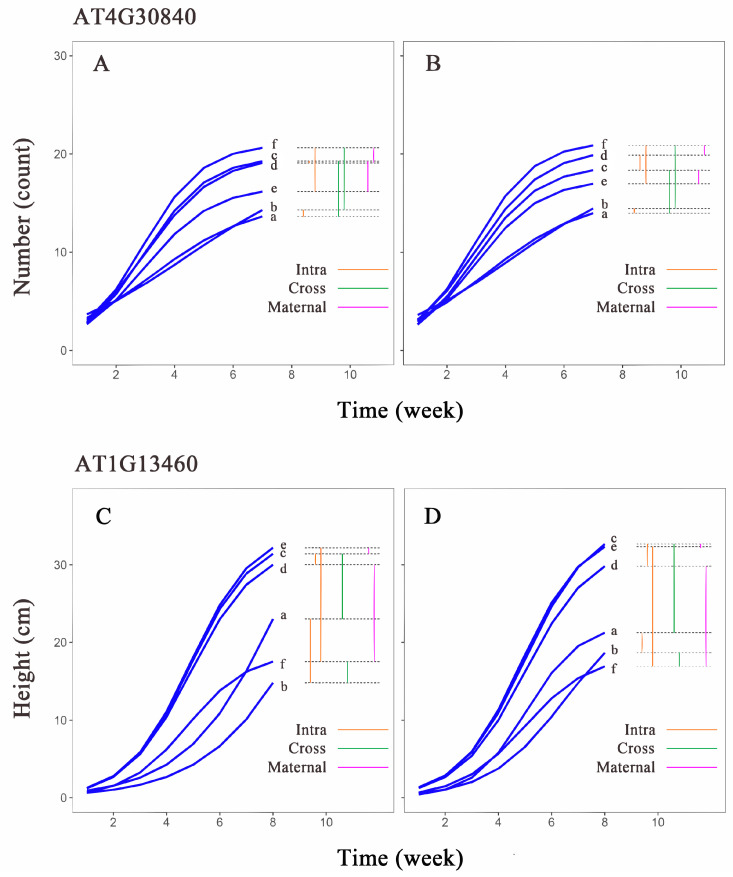
Growth curves estimated for two genotypes of hub genes in *Arabidopsis thaliana*. Growth curves estimated for (**A**) aa and (**B**) AA genotypes of *AT4G30840* in leaf number trait group; (**C**) aa and (**D**) AA genotypes of *AT1G13460* in plant height group. Horizontal coordinates are measurement time points, and vertical coordinates are phenotypic value units. a–f are each phenotypic combination (a, H_1_; b, L_1_; c, H_1_H_2_; d, H_1_L_2_; e, L_1_H_2_; f, L_1_L_2_). Intra (orange) represents intergenerational phenotypic plasticity; Cross (green) represents transgenerational phenotypic plasticity; and Maternal (purple) represents maternal effects.

Comparing the leaf number growth curves of the *AT4G30840* gene in different populations of *Arabidopsis thaliana* (Figure 7A,B), we found that the growth curve differences between the aa genotype in H_1_ (curve a) and H_1_H_2_ (curve c) were similar to the differences between L_1_ (curve b) and L_1_L_2_ (curve f) under two different light conditions. This suggests that the aa genotype exhibits similar transgenerational plasticity under both light conditions, while the AA genotype shows greater transgenerational plasticity under low light compared to high light. In the first-generation population, both genotypes H_1_ (curve a) and L_1_ (curve b) displayed minimal growth differences, indicating low within-generation plasticity in this population. The within-generation plasticity of the L_1_H_2_ and L_1_L_2_ genotypes was higher than that of the second-generation populations, H_1_H_2_ and H_1_L_2_. The aa genotype also exhibited minimal curve differences between H_1_H_2_ (curve c) and H_1_L_2_ (curve d), whereas the growth curve of L_1_H_2_ (curve e) differed significantly from that of L_1_L_2_ (curve f). The AA genotype showed a similar pattern of differences, but the differences were less pronounced compared to the aa genotype. The differences between the AA genotype in H_1_L_2_ (curve d) and L_1_L_2_ (curve f) were similar to the differences between H_1_H_2_ (curve c) and L_1_H_2_ (curve e), while the differences between H_1_H_2_ (curve c) and L_1_H_2_ (curve e) in the aa genotype were greater than the differences between H_1_L_2_ (curve d) and L_1_L_2_ (curve f). This suggests that the aa genotype is more susceptible to maternal environmental influences. 

Comparing the plant height growth curves of the *AT1G13460* gene in different populations (Figure 7C,D), we found that the growth curves of H_1_ (curve a) and H_1_H_2_ (curve c) of the two genotypes widely differed from each other; however, the differences between L_1_ (curve b) and L_1_L_2_ (curve f) were small, which indicated that this gene exhibits similar transgenerational plasticity across different genotypes. Notably, the AA genotype demonstrates significantly different transgenerational plasticity under two different light conditions, compared to the aa genotype. Between the first-generation populations, the differences in the growth curves of the two genotypes were different, and the differences between the aa genotype for H_1_ (curve a) and L_1_ (curve b) were much larger than those of the AA genotype. Between the second-generation populations, the growth curves of the two genotypes for L_1_H_2_ (curve e) differed more from those of L_1_L_2_ (curve f). The difference between the curves of H_1_H_2_ (curve c) and H_1_L_2_ (curve d) was smaller, which indicated that the plasticity of the aa genotypes was much larger than that of the AA genotypes between the first-generation populations, and the plasticity between the two genotypes for L_1_H_2_ and L_1_L_2_ was larger than that between H_1_H_2_ and H_1_L_2_ in the second-generation population. The differences between AA genotypes for H_1_L_2_ (curve d) and L_1_L_2_ (curve f) were considerable; the differences between H_1_H_2_ (curve c) and L_1_H_2_ (curve e) were small. The growth curves of the aa genotypes showed a similar pattern, but the differences were not as large as those of the AA genotypes, which suggests that differences in the maternal environments of the genes have little effect on the offspring under high-light conditions but a strong effect on offspring in low-light environments. 

## 4. Discussion

Leaf number and plant height are important phenotypic characteristics in *thaliana*, and light is an important environmental factor affecting phenotype. To determine how light affects the leaf number and plant height phenotypes, we conducted an analysis of the association between phenotype and genotype as an important tool to answer this question from the genetic point of view. Compared with genome-wide association studies (GWAS) for static traits, this study used functional mapping to analyze the obtained dynamic phenotypic data to locate a series of genes associated with leaf number and plant height under high- and low-light conditions. By comparing the significant genes in the different light-treated groups in different generations and quantifying their genetic effects, we attempted to explain the genetic mechanisms underlying the transgenerational plasticity of *thaliana* in response to light conditions.

Our observations showed that the first-generation population had a higher number of outliers than the second-generation population, with the majority occurring during the middle stages of growth. This indicated that the first-generation population had a higher proportion of individuals with growth patterns that significantly deviated from those of the overall population. We speculate that the presence of growth rate inflection points during the middle stages amplified the individual growth differences, resulting in a higher distribution of outliers during this period. This also affected the kurtosis and skewness coefficients of the phenotypic data. Comparisons of the mean phenotype values between the different treatments (Figure 2) revealed that *Arabidopsis* leaves exhibited transgenerational adaptive growth when subjected to low-light treatment in the first generation and were subsequently faced with low-light conditions again, consistent with previous findings reported by Robinson [8]. Corresponding to the increased leaf count in the L_1_L_2_ group, the plant height of the L_1_L_2_ group was significantly lower than that of the L_1_H_2_ group. *Thaliana* tends to mitigate adverse environmental conditions by differentially modulating leaf elongation in response to low light environments [29]. This suggests that the species may allocate more biomass to leaf growth rather than to plant elongation. In ample light conditions, no significant difference was found in plant height between the H_1_H_2_ and L_1_H_2_ groups, indicating the sensitivity of *thaliana* to changes in the light environment, with transgenerational effects typically being activated only in the face of environmental stress.

We found a large number of genes encoding RING/U-box superfamily proteins that are associated with E3 ubiquitin ligases from the description of significant genes for each phenotypic combination. The ubiquitin-mediated degradation of the proteasome is one of the major mechanisms of the post-translational regulation of gene expression and protein quality control in eukaryotes and is involved in a wide range of cellular activities, including biotic and abiotic stress responses, signal transduction, transcriptional regulation, DNA repair, and organelle biogenesis [30,31]. Protein ubiquitination is mainly mediated via a three-step enzymatic process, namely, ubiquitin-activating enzyme E1, ubiquitin-conjugating enzyme E2, and ubiquitin ligase E3. E3 ubiquitin ligases, as the largest family of the three enzymes, can be classified into different categories based on their structure, function, and substrate specificity: RING, HECT (homologous to the E6AP carboxyl terminus), CRL (Cullin-RING ligases), and U-box [32]. RING/U-box E3 ubiquitin ligases are involved in various plant developmental processes and stress signal transduction; for example, U-box proteins interfere with abscisic acid response in *thaliana* [33], and the RING-type E3 ubiquitin ligase COP1 (constitutively photomorphogenic 1) protein plays a central switching role in the light regulation of *thaliana* seedling development [34]. The genes encoding F-box proteins were found in the traits H_1_ and L_1_ for both plant height and number of leaves. The F-box motif of F-box proteins is located at the N-terminus, whereas the C-terminal region usually has one or more structural domains for protein interactions, resulting in high variability [35,36]. Based on the C-terminal structural domains, the F-box family of genes can be categorized into several subfamilies, such as leucine-rich repeat (LRR), beta-transducin (WD40) repeat, tetraploid repeat (G-TPR), or Kelch repeat [37,38]. The F-box protein sequence variability leads to functional diversity, and F-box proteins are mainly involved in biological processes such as growth and development, cellular protein degradation, reproduction, embryogenesis, seed emergence, biotic and abiotic stress responses, endogenous hormone signaling, and senescence as components of the formation of the Skp-cullin-f-box (SCF) complex [39,40,41,42,43,44]. Notably, we also identified the methyltransferase MT-A70 family protein encoded by *AT1G19340*, a mammalian METTL4 direct homolog [45]. The enzymatic covalent modification of RNA is an important epigenetic mechanism that finely and plasticly regulates a wide range of cellular activities in eukaryotes [46]. Among them, N6-methyladenosine (m6A) methylation is one of the common RNA modifications, and MT-A70 family proteins can mediate the catalytic mechanism of m6A methylation [47], which is involved in the plasticity in response to different environments.

The analysis of the effect network diagram under low-light conditions indicates that the phenotypic response to low light involves the regulatory effects of multiple genes. The hub gene, *AT1G15040*, encodes a class I glutamine amidotransferase (GAT1), and studies have shown that GAT1 has a significant inhibitory function on branching and is involved in branch regulation under nitrogen stress. Mutants of GAT exhibit a quicker manifestation of increased leaf and flower phenotypes [48]. In this study, the L_1_L_2_ population also exhibited an increased number of leaves and a dwarf plant phenotype, suggesting a correlation with the gene experiencing more inhibitory effects in the network diagram. The increase in branching to some extent leads to an increase in the number of leaves. The hub gene, *AT1G13460,* is associated with the response to low-light stimulus (GO:0009645). This gene encodes a regulatory B subunit of protein phosphatase 2A (PP2A). PP2A is a crucial and abundant serine/threonine phosphatase in eukaryotic cells, with the B subunit primarily controlling substrate recognition [49]. PP2A can counteract various kinases involved in cell growth, proliferation, apoptosis, cytoskeletal dynamics, and stress responses. It plays a significant role in regulating plant development and responding to various stresses [50]. In addition to mutually inhibiting each other, these two hub genes interact with the linking gene, *AT2G47670*. *AT2G47670* has been shown to be highly co-expressed with ATL54 and is involved in programmed cell death during secondary wall biosynthesis and lignification [51]. Additionally, the hub gene, *AT2G46495,* encodes a RING/U-box superfamily protein, which is believed to have potential phosphorylation hotspot regions [52]. Protein phosphorylation, as a critical post-translational modification, influences numerous aspects of dynamic cellular behavior. According to network analysis, *AT2G46495* directly inhibits the hub gene, *AT2G47420*. *AT2G47420* encodes ADENOSINE DIMETHYLTRANSFERASE 1A (DIM1A), an RNA-binding (RRM/RBD/RNP motifs) family protein (RBP) involved in ribosome biosynthesis, which has been found to be associated with epidermal cell formation [53]. Thus, it is evident that complex interactions between genes ultimately influence phenotype formation.

The GO analysis of the low-light group for leaf number indicates that the gene *AT4G09040* (GO:0009641), associated with shade avoidance, also encodes an RBP. This protein regulates the transcription of corresponding genes in the organism by controlling mRNA localization, thereby modulating expression levels. *AT4G09040* thus conducts the corresponding protein modifications and subcellular localization to improve plant resistance to environmental and other stresses [54]. The GO analysis of the high-light environment similarly indicates a large number of genes involved in hormone regulation and signal transduction processes. For instance, it was found that in the high-light leaf group, the gene AT4G36740, which is associated with the regulatory entry of the gibberellic-acid-mediated signaling pathway (GO:0009937), encodes a gibberellic acid (GA) homeostatic regulator homeobox protein 40 (HB40) [55]. HB40 directly activates the NAC (NAM, ATAF1/2, CUC2) transcription factor JUB1(JUNGBRUNNEN1) [56], which inhibits GA synthesis and genes encoding C19-GA-inactivating enzymes (GA 2-oxidases GA2ox2 and GA2ox6). HB40-overexpressing plants grew to exhibit the typical associated GA-deficient traits. In the high-light plant height group, it was found that *AT4G36090* in the gene related to the cell surface receptor signaling pathway (GO:0007166) encodes a 2-oxoglutarate and Fe (II)-dependent dioxygenase family protein (2OG-Fe (II) oxygenase family protein, 2ODDs). The family proteins, 2ODDs, are involved in a variety of important metabolic pathways, including melatonin metabolism [57,58], the anabolic metabolism of important phytohormones [59], and the biosynthesis of secondary metabolites [60] and can directly affect plant growth, development, and the stress response [61]. 

The genotype growth curve indicates that a substantial portion of the large plasticity difference between the two genotypes of *AT4G30840* is due to the growth disparity in the L_1_H_2_ generation. According to functional localization results, the genotype difference is associated with the significance of the gene; therefore, we speculate that *AT4G30840* is significantly expressed under L_1_H_2_ conditions. *AT4G30840* encodes a Transducin/WD40 repeat-like superfamily protein, and WD40 proteins act as multifunctional scaffold proteins, regulating protein–protein interactions in various cellular processes, such as plant stress and hormone responses [62]. 

In contrast, the growth gap between the two genotypes in L_1_ and L_1_L_2_ consistently increased over time. *AT1G13460* encodes the B subunit of the protein phosphatase PP2A, which, in concert with kinases, is often associated with signaling. The more pronounced genotypic disparity of *AT1G13460* in low-light environments, compared with high-light environments, corresponds to the finding that this gene is associated with GO entries regarding the response to low-light-intensity stimuli. Additionally, the signaling pathway involved in PP2A may be equally active in the mid-growth phase of H_1_ populations.

We found that some genes are involved in the response of leaf number and plant height to the same environment from the combined study of the two traits, which corresponds to the strong correlation between the traits. Moreover, we found that these genes are similar in the types of proteins they encode, mostly protein kinases, binding proteins, and transcription factors. For example, *AT4G35390* encodes the DNA-binding protein of the negative GA feedback cis-acting sequence (AT-hook protein of GA feedback 1, ACF1), which binds to the negative GA feedback cis-acting sequence of AtGA3ox1 (gibberellin 3-oxidase 1 in *Arabidopsis*). GA 3-oxidase (GA3ox) is the last part catalyzed into physiologically active GA, and only AtGA3ox1 in the *Arabidopsis thaliana* AtGA3ox family is regulated by negative GA feedback [63]. AtGA3ox1 is mainly associated with plant nutrient growth [64] and is expressed in seedlings, leaves, stems, floral tips, and flowers [65]. This suggests that ACF1 is involved in the regulation of GA homeostasis and, thus, affects the number of leaves and plant height. In addition, more duplicated genes encode protein kinases (PK). PK, as one of the largest and most diverse plant protein superfamilies involved in biological processes, including mitosis and cytoplasmic division [66,67], cell growth and elongation [68], organogenesis [69], photosynthesis [70], and hormonal responses [71], plays a key role in the plant phenotypic response to environmental changes.

We identified the genes involved in the response of the leaf number and plant height phenotypes to heterogeneous light environments across different generations, and we observed the transgenerational adaptive growth of the leaf number phenotype under low-light stress. Further research could involve expanding the sample size and incorporating additional generations to more accurately examine the genetic bases of plasticity and evaluate the consistency of this transgenerational adaptation. Additionally, because plants are influenced by various environmental factors during growth, in reality, other factors such as humidity, population density, and nutrient levels could be introduced to investigate the changes in the plasticity of plants under multiple stressors.

## 5. Conclusions

We used *thaliana* as the study material, then gathered phenotypic data regarding leaf number and plant height under high- and low-light conditions from two generations. Using the obtained genotype data, a functional mapping model was used to locate a series of significant SNPs. We observed similarities and differences in the significant genes among the different generations, and a gene-encoding methyltransferase linked to epigenetics was discovered. Subsequently, we constructed a genetic effect network and performed GO analysis to explore the gene functions annotated to the candidate genes. The results revealed complex interactions among these genes, primarily associated with signaling transduction, hormone pathways, light response, and organ development regulation. Further analysis of the significant genes *AT4G30840* and *AT1G13460*’s genetic effects unveiled their distinct involvement in the regulation of light response processes. We also observed variations in their impact on phenotypic plasticity under different light treatments, providing evidence for transgenerational inheritance and the intricate relationship between light-induced plasticity and phenotype. Our study offers insights into this process at the genetic level and provides clues for further exploration of the genetic mechanisms underlying transgenerational plasticity in *thaliana*’s response to light conditions.

## Figures and Tables

**Table 1:** Abbreviations for plasticity group name.

Category	Phenotypic Analysis Group	Abbreviation
Intragenerational plasticity	High-light and low-light	H_1_ and L_1_
	High-light–high-light and high-light–low-light	H_1_H_2_ and H_1_L_2_
	Low-light–high-light and low-light–low-light	L_1_H_2_ and L_1_L_2_
Cross-generational plasticity	High-light and high-light–high-light	H_1_ and H_1_H_2_
	Low-light and low-light–low-light	L_1_ and L_1_L_2_
Maternal effect	High-light–high-light and low-light–high-light	H_1_H_2_ and L_1_H_2_
	High-light–low-light and low-light–low-light	H_1_L_2_ and L_1_L_2_

**Table 2:** Summary of significant genes for leaf number and plant height traits of *Arabidopsis thaliana* under high-light treatment.

Category	Gene ID	Position	Chr	Alle	Variation Type	Gene Description
Developmental regulation	*AT4G30840*	15023742	4	T/T	upstream_gene	Transducin/WD40 repeat-like superfamily protein
	*AT4G34220*	16388827	4	A/A	upstream_gene	Leucine-rich repeat protein kinase family protein
	*AT4G35165*	16739054	4	C/C	downstream_gene	Egg cell-secreted-like protein (DUF1278)
	*AT4G35370*	16812606	4	A/A	upstream_gene	Transducin/WD40 repeat-like superfamily protein
	*AT4G35390*	16829046	4	C/C	upstream_gene	AT-hook protein of GA feedback 1
	*AT4G35840*	16982215	4	C/C	synonymous	RING/U-box superfamily protein
	*AT4G35860*	16989499	4	T/T	upstream_gene	GTP-binding 2
	*AT4G35900*	17005228	4	G/G	missense	Basic-leucine zipper (bZIP) transcription factor family protein
	*AT4G35910*	17010429	4	G/G	synonymous	Adenine nucleotide alpha hydrolases-like superfamily protein
	*AT4G35950*	17026971	4	A/A	upstream_gene	RAC-like 6
	*AT4G36080*	17065428	4	G/G	synonymous	Phosphotransferases/inositol or phosphatidylinositol kinase
	*AT4G36160*	17118705	4	T/T	upstream_gene	NAC domain containing protein 76
	*AT4G36630*	17273803	4	C/C	synonymous	Vacuolar sorting protein 39
Stress response	*AT2G14210*	6018220	2	T/T	upstream_gene	AGAMOUS-like 44
	*AT4G35920*	17013293	4	C/C	splice_region&synonymous	PLAC8 family protein
	*AT4G36150*	17107236	4	C/C	missense	Disease resistance protein (TIR-NBS-LRR class) family
	*AT4G36140*	17109354	4	T/T	upstream_gene	Disease resistance protein (TIR-NBS-LRR class)
	*AT4G37640*	17688360	4	G/G	upstream_gene	Calcium ATPase 2
Transcriptional regulation	*AT4G35890*	16995463	4	C/C	upstream_gene	Winged-helix DNA-binding transcription factor family protein
	*AT4G36590*	17265352	4	C/C	upstream_gene	MADS-box transcription factor family protein
	*AT4G36650*	17285093	4	T/T	synonymous	Plant-specific TFIIB-related protein
Anabolism	*AT4G35650*	16910021	4	T/T	3_prime_UTR	Isocitrate dehydrogenase III
	*AT4G35640*	16911160	4	A/A	upstream_gene	Serine acetyltransferase 3;2
	*AT4G36090*	17079871	4	T/T	synonymous	Oxidoreductase, 2OG-Fe(II) oxygenase family protein
	*AT4G36250*	17151190	4	A/A	synonymous	Aldehyde dehydrogenase 3F1
	*AT4G36360*	17183722	4	T/T	upstream_gene	Beta-galactosidase 3
Other	*AT2G14310*	6070613	2	A/A	upstream_gene	Pseudo
	*AT4G35660*	16912804	4	T/T	missense	Selection/upkeep of intraepithelial T-cell protein, putative (DUF241)
	*AT4G35837*	16984099	4	C/C	upstream_gene	Hypothetical protein
	*AT4G36120*	17098217	4	C/C	upstream_gene	Filament-like protein (DUF869)
	*AT4G35980*	17032032	4	T/T	upstream_gene	Uncharacterized protein
	*AT4G36197*	17129567	4	T/T	upstream_gene	tRNA-Glu
	*AT4G36648*	17286112	4	C/C	upstream_gene	ncRNA

**Table 3:** Summary of significant genes for leaf number and plant height traits in *Arabidopsis thaliana* under low-light treatment.

Category	Gene ID	Position	Chr	Alle	Variation Type	Gene Description
Developmental regulation	*AT1G16440*	5616819	1	G/G	missense	Root hair specific 3
	*AT2G27520*	11762957	2	T/T	synonymous	F-box and associated interaction domains-containing protein
Stress response	*AT1G16670*	5694727	1	G/G	upstream_gene	Protein kinase superfamily protein
	*AT2G27580*	11777295	2	A/A	5_prime_UTR	A20/AN1-like zinc finger family protein
	*AT2G28060*	11952520	2	T/T	upstream_gene	5′-AMP-activated protein kinase beta-2 subunit protein
Transcriptional regulation	*AT2G27700*	11818626	2	C/C	upstream_gene	Eukaryotic translation initiation factor 2 family protein/eIF-2 family protein
	*AT2G27880*	11878388	2	A/A	downstream_gene	Argonaute family protein
Anabolism	*AT2G18640*	8086595	2	T/T	upstream_gene	Geranylgeranyl pyrophosphate synthase 4
	*AT2G27570*	11775564	2	G/G	stop_gained	P-loop containing nucleoside triphosphate hydrolases superfamily protein
	*AT2G27650*	11801094	2	G/G	upstream_gene	Ubiquitin carboxyl-terminal hydrolase-related protein
Other	*AT1G15610*	5371155	1	G/G	missense	Uncharacterized protein
	*AT2G27740*	11821497	2	G/G	upstream_gene	RAB6-interacting golgin (DUF662)
	*AT2G28180*	12017798	2	C/C	upstream_gene	Cation/hydrogen exchanger family protein

## Data Availability

Data and analysis codes are available for download from https://github.com/fssq811/Arabidopsis/tree/master/code (accessed on 10 June 2024).

## Supplementary Materials

The following supporting information can be downloaded at: https://www.mdpi.com/article/10.3390/cimb46080482/s1: Figure S1: Box plot of *Arabidopsis thaliana* leaf number and plant height for the H_1_ and L_1_ populations. Figure S2: Box plot of *Arabidopsis thaliana* leaf number and plant height for the H_1_H_2_ and H_1_L_2_ populations. Figure S3: Box plot of *Arabidopsis thaliana* leaf number and plant height for the L_1_H_2_ and L_1_L_2_ populations. Figure S4: Manhattan plots of significant loci in different populations of *Arabidopsis thaliana* according to functional mapping analysis. Figure S5: Quantile-quantile plots of *p*-values for different populations of *Arabidopsis thaliana*. Figure S6: Genetic network diagrams of different *Arabidopsis thaliana* populations. Table S1: Analysis of variation in phenotypic trait of leaf number. Table S2. Analysis of variance for phenotypic trait of plant height. Table S3: Significant loci of leaf number trait of *Arabidopsis thaliana* in H_1_ generation. Table S4: Significant loci of leaf number trait of *Arabidopsis thaliana* in L_1_ generation. Table S5: Significant loci of leaf number trait of *Arabidopsis thaliana* in H_1_H_2_ generation. Table S6: Significant loci of leaf number trait of *Arabidopsis thaliana* in H_1_L_2_ generation. Table S7: Significant loci of leaf number trait of *Arabidopsis thaliana* in L_1_H_2_ generation. Table S8: Significant loci of leaf number trait of *Arabidopsis thaliana* in L_1_L_2_ generation. Table S9: Significant loci of plant height trait of *Arabidopsis thaliana* in H_1_ generation. Table S10: Significant loci of plant height trait of *Arabidopsis thaliana* in L_1_ generation. Table S11: Significant loci of plant height trait of *Arabidopsis thaliana* in H_1_H_2_ generation. Table S12: Significant loci of plant height trait of *Arabidopsis thaliana* in H_1_L_2_ generation. Table S13: Significant loci of plant height trait of *Arabidopsis thaliana* in L_1_H_2_ generation. Table S14: Significant loci of plant height trait of *Arabidopsis thaliana* in L_1_L_2_ generation.
