# Supplementary material for: Mapping the Influence of Light Intensity on the Transgenerational Genetic Architecture of Arabidopsis thaliana
Source: Curr Issues Mol Biol. 2024 Jul 29;46(8):8148–69. doi: 10.3390/cimb46080482 (PMC11352948; doi:10.3390/cimb46080482)
Supplement: Supplementary file 1 [file cimb-46-00482-s001.zip › cimb-3093396-supplementary.pdf]

Supplementary Figures

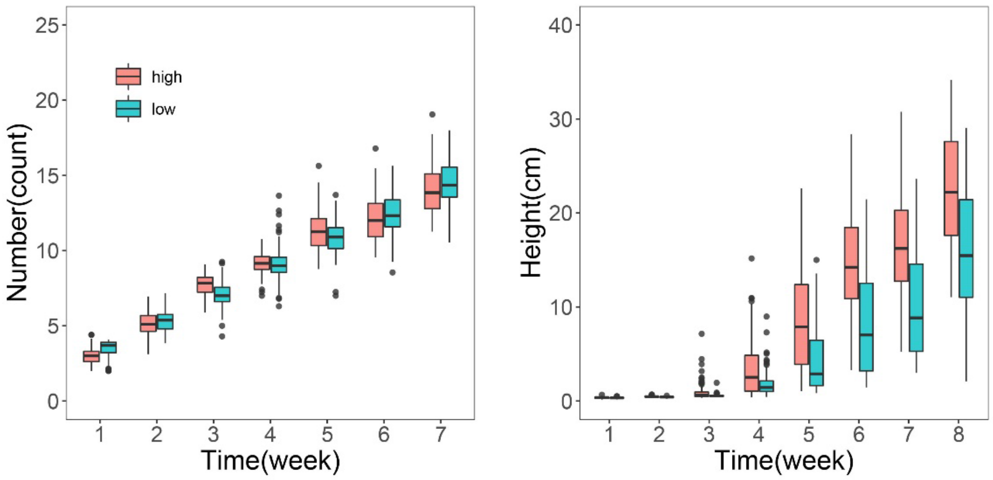

(a) (b)

Figure S1. (a) The box plot of *Arabidopsis thaliana* leaf number for the H_1 and L_1 populations; **(b)** The box plot of *Arabidopsis thaliana* plant height for the H_1 and L_1 populations. The abbreviations for the populations can be found in Section 2.1. The x-axis represents the measurement time points, and the y-axis represents the phenotypic values. The red color indicates the high-light environment, while the green color represents the low-light environment. The line segments at either end represent the maximum and minimum values, while the upper and lower edges of the box represent the upper and lower quartiles (the 25th and 75th percentiles, respectively, when all values in the sample are arranged in ascending order). The thick line in the middle of the box represents the median (the 50th percentile when all values in the sample are arranged in ascending order). The dots represent outliers.

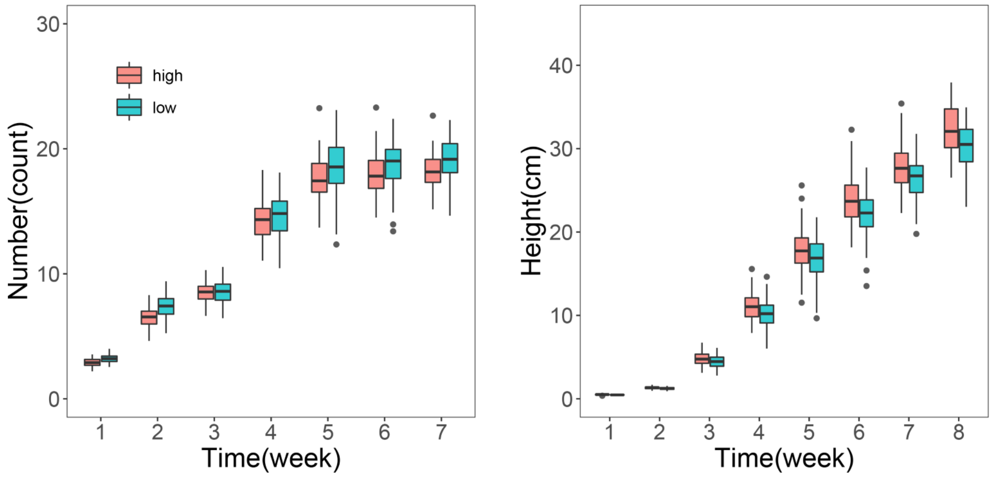

(a) (b)

Figure S2. (a) The box plot of *Arabidopsis thaliana* leaf number for the H_1H_2 and H_1L_2 populations; **(b)** The box plot of *Arabidopsis thaliana* plant height for the H_1H_2 and H_1L_2 populations. The abbreviations for the populations can be found in Section 2.1. The x-axis represents the measurement time points, and the y-axis represents the phenotypic values. The red color indicates the high-light environment, while the green color represents the low-light environment. The line segments at either end represent the maximum and minimum values, while the upper and lower edges of the box represent the upper and lower quartiles (the 25th and 75th percentiles, respectively, when all values in the sample are arranged in ascending

order). The thick line in the middle of the box represents the median (the 50th percentile when all values in the sample are arranged in ascending order). The dots represent outliers.

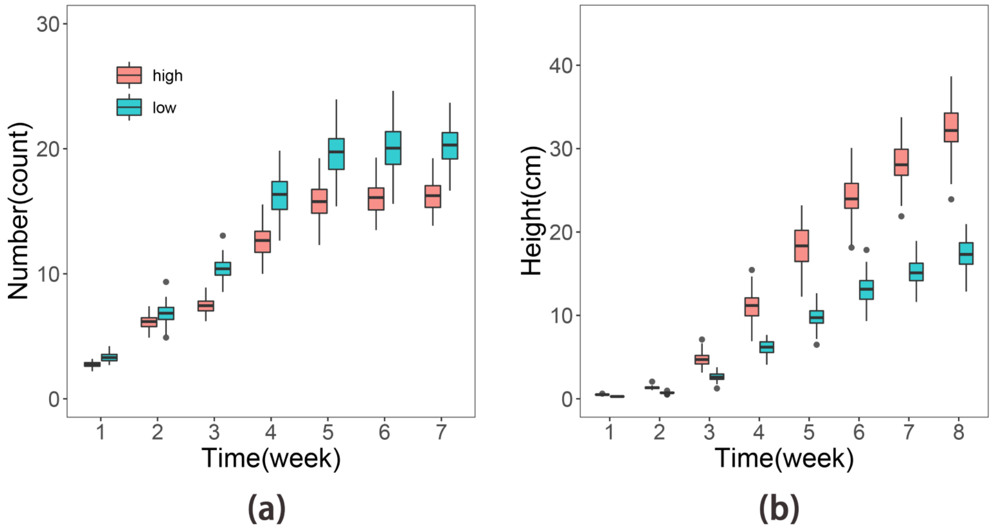

Figure S3. (a) The box plot of *Arabidopsis thaliana* leaf number for the L_1H_2 and L_1L_2 populations; **(b)** The box plot of *Arabidopsis thaliana* plant height for the L_1H_2 and L_1L_2 populations. The abbreviations for the populations can be found in Section 2.1. The x-axis represents the measurement time points, and the y-axis represents the phenotypic values. The red color indicates the high-light environment, while the green color represents the low-light environment. The line segments at either end represent the maximum and minimum values, while the upper and lower edges of the box represent the upper and lower quartiles (the 25th and 75th percentiles, respectively, when all values in the sample are arranged in ascending order). The thick line in the middle of the box represents the median (the 50th percentile when all values in the sample are arranged in ascending order). The dots represent outliers.

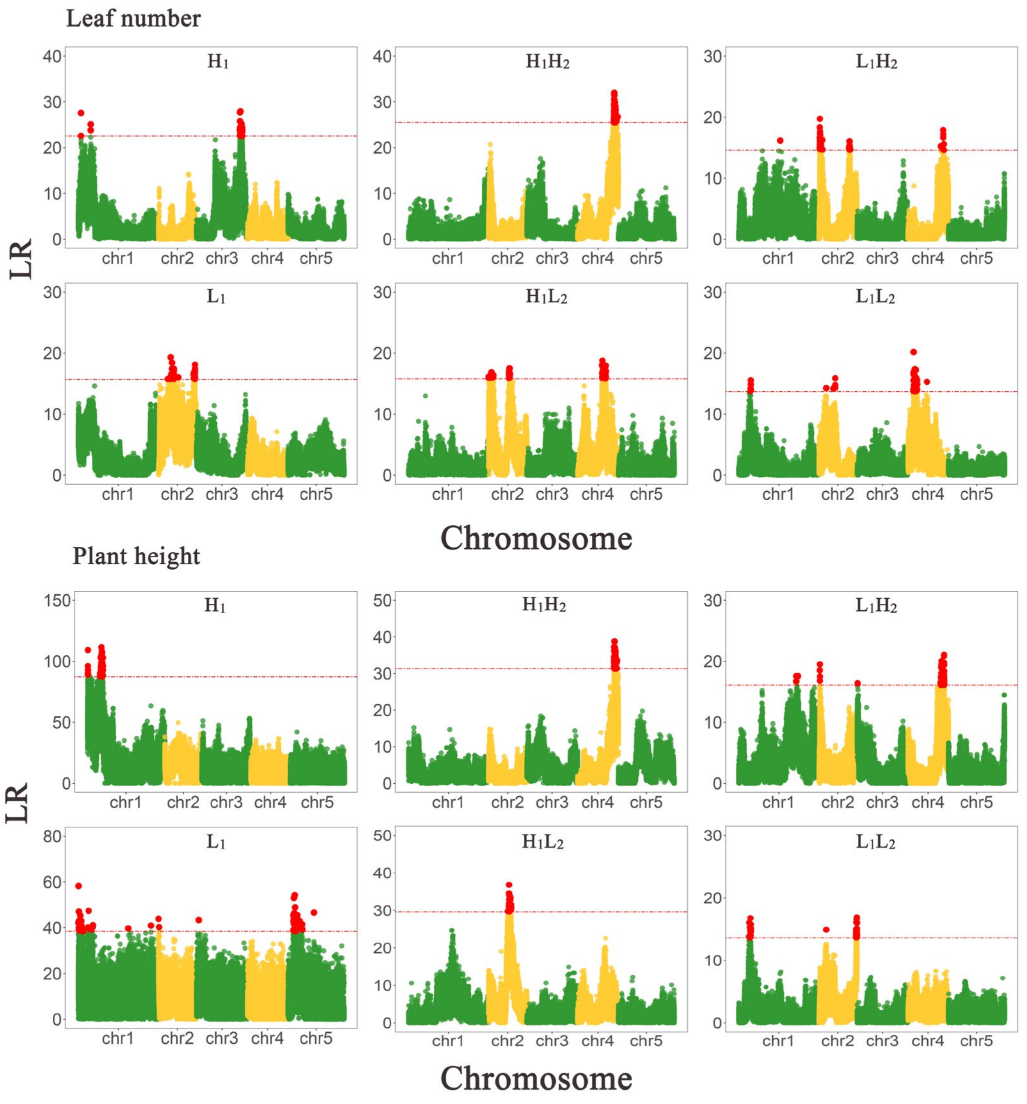

Figure S4. Manhattan plots of significant loci in different populations of *Arabidopsis thaliana* according to functional mapping analysis. The upper part is the leaf number, and the lower part is the plant height. Refer to Section 2.1 for the meaning of the abbreviations of the populations; the horizontal coordinate is the chromosome position where the SNP locus is located; the vertical coordinate is the likelihood ratio statistic of the SNP locus; the red dashed line is the threshold obtained from the 1000 permutation assay; and the red loci on top of it are the SNP loci significantly correlated with the phenotype.

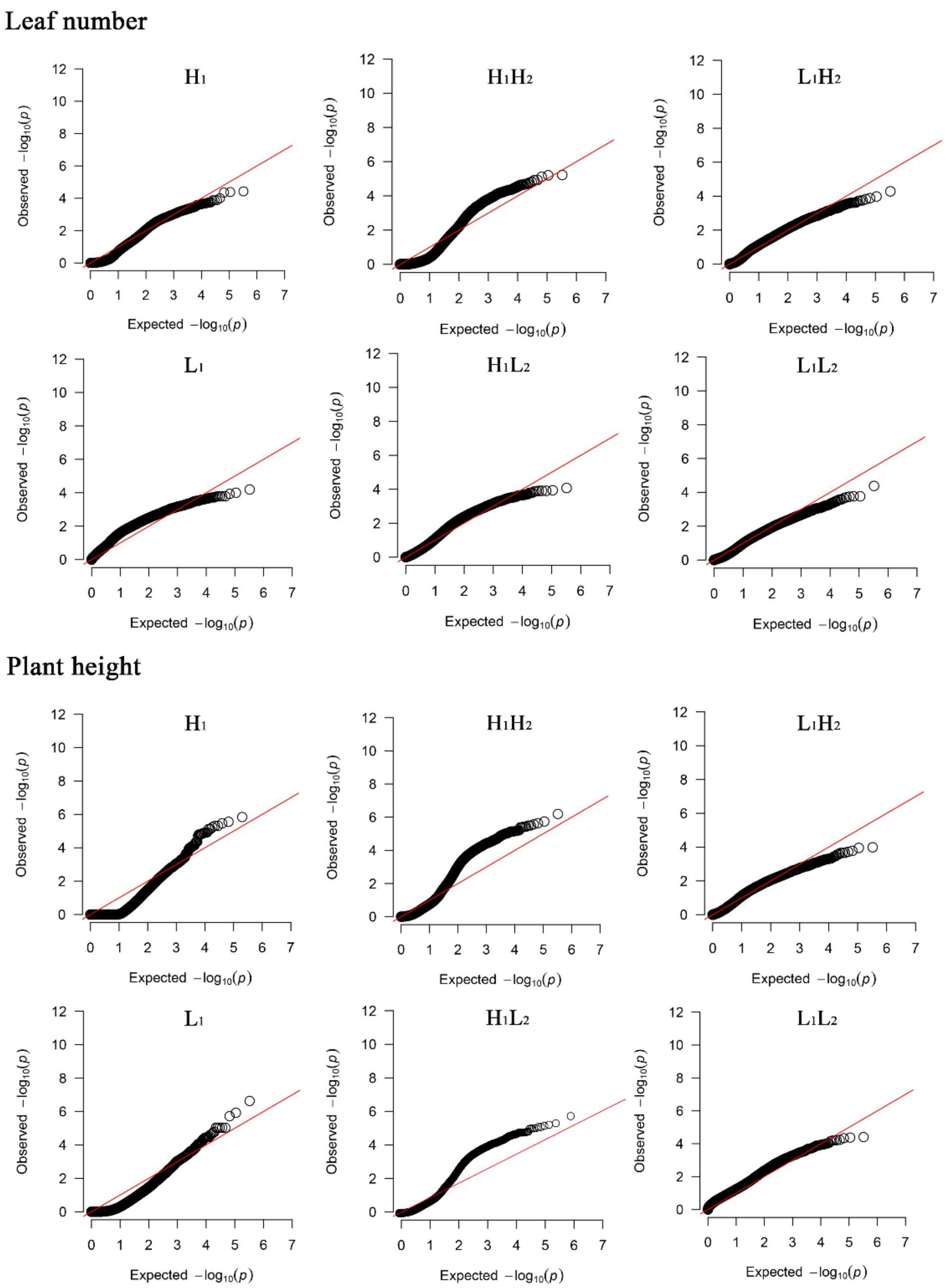

Figure S5. Quantile–quantile plots of p-values for different populations of *Arabidopsis thaliana.* Leaf number is depicted in the upper panel, while plant height is shown in the lower panel. Refer to Section 2.1 for the meaning of the abbreviations of the populations; the x-axis represents the expected distribution, and the y-axis represents the observed distribution.

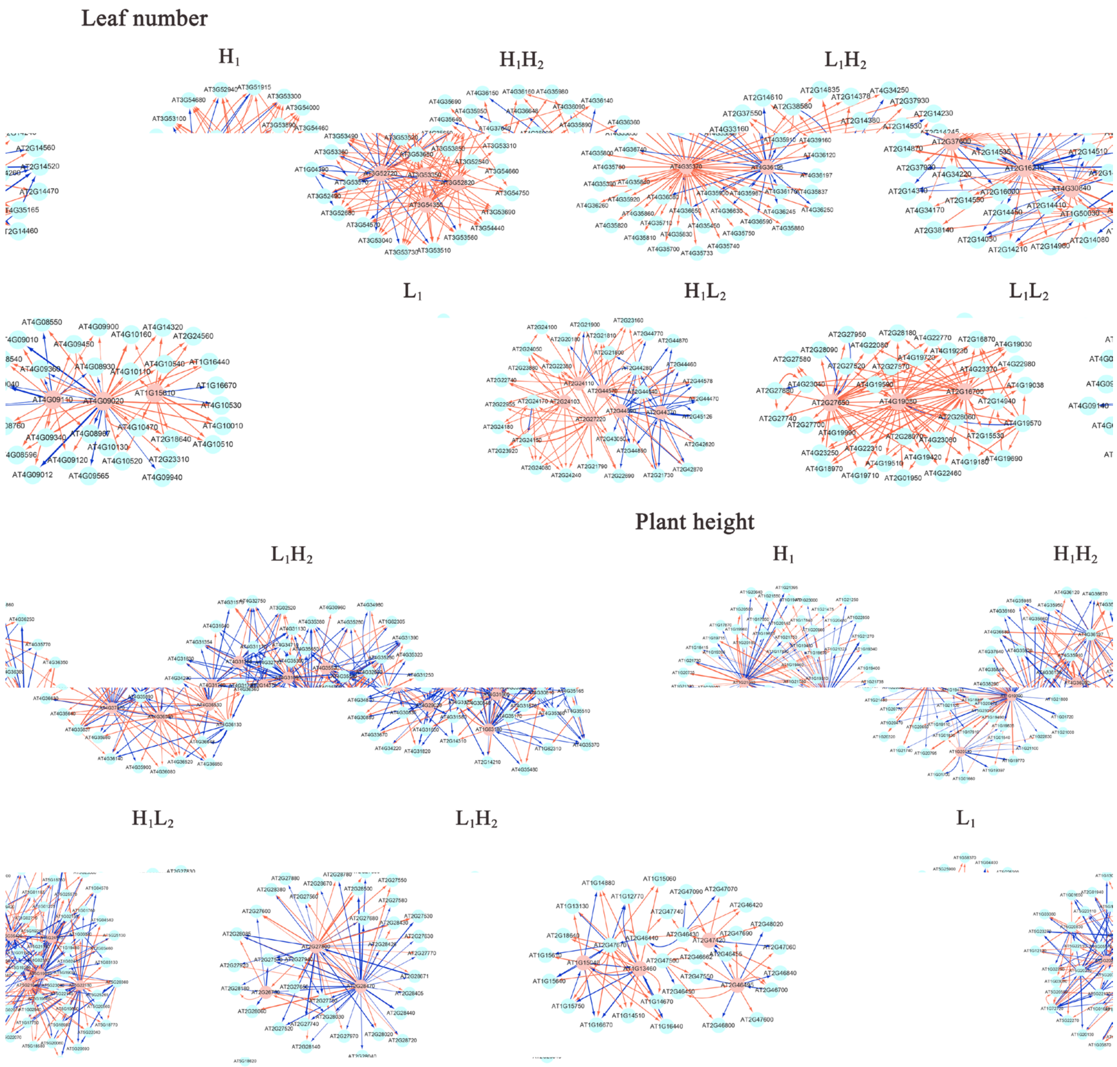

Figure S6. Genetic network diagrams of different *Arabidopsis thaliana* populations. Refer to Section 2.1 for the meaning of the abbreviations of the populations; pink nodes represent hub genes. Red arrows indicate activation, while blue arrows indicate inhibition. The thicker the arrow, the greater the impact; conversely, the thinner the arrow, the smaller the impact.

Supplementary Tables

Table S1. Analysis of variation in leaf number phenotypic traits.

Population	Time(week)	Mean	SD	Range	CV	Kurtosis	Skewness
H_1	1	3.00	0.55	2.40	0.18	-0.06	0.51
	2	5.19	0.78	3.83	0.15	-0.33	0.11
	3	7.72	0.69	3.22	0.09	0.11	-0.42
	4	9.14	0.73	3.75	0.08	0.51	-0.37
	5	11.36	1.46	6.86	0.13	-0.03	0.55
	6	12.05	1.56	7.24	0.13	-0.14	0.56
	7	14.07	1.57	7.79	0.11	0.21	0.59
L_1	1	3.50	0.53	2.10	0.15	1.14	-1.35
	2	5.35	0.72	3.31	0.13	-0.37	0.21
	3	7.03	0.88	4.95	0.13	0.54	-0.15
	4	9.18	1.25	7.35	0.14	1.74	0.84
	5	10.94	1.25	6.70	0.11	0.71	-0.20
	6	12.42	1.40	7.08	0.11	0.07	0.09
	7	14.49	1.43	7.45	0.10	0.01	0.07
H_1H_2	1	2.88	0.31	1.35	0.11	-0.77	-0.17
	2	6.55	0.73	3.67	0.11	-0.17	0.04
	3	8.54	0.73	3.67	0.09	-0.16	0.04
	4	14.24	1.44	7.25	0.10	-0.46	0.23
	5	17.59	1.80	9.55	0.10	-0.01	0.12
	6	18.01	1.61	8.80	0.09	0.18	0.42
	7	18.17	1.41	7.49	0.08	-0.07	0.17
H_1L_2	1	3.20	0.31	1.45	0.10	-0.41	0.22
	2	7.37	0.88	4.15	0.12	-0.31	-0.11
	3	8.54	0.88	4.10	0.10	-0.35	-0.08
	4	14.69	1.69	7.65	0.12	-0.38	-0.24
	5	18.46	2.19	10.75	0.12	-0.06	-0.38
	6	18.77	1.81	9.00	0.10	0.21	-0.53
	7	19.18	1.67	7.65	0.09	-0.33	-0.37
L_1H_2	1	2.76	0.20	1.00	0.07	-0.28	-0.18
	2	6.18	0.60	2.51	0.10	-0.49	0.14
	3	7.46	0.61	2.69	0.08	-0.41	0.25
	4	12.57	1.19	5.55	0.09	-0.58	-0.04
	5	15.74	1.41	6.95	0.09	-0.59	-0.18
	6	16.04	1.26	5.80	0.08	-0.52	-0.07
	7	16.21	1.14	5.40	0.07	-0.45	-0.07
L_1L_2	1	3.32	0.37	1.51	0.11	-0.65	0.35
	2	6.86	0.74	4.45	0.11	0.63	0.16
	3	10.43	0.77	4.50	0.07	0.62	0.26
	4	16.31	1.38	7.20	0.08	-0.40	-0.13
	5	19.74	1.73	8.55	0.09	-0.32	0.08

	6	20.10	1.67	9.03	0.08	-0.35	0.11
	7	20.25	1.48	7.03	0.07	-0.47	-0.08

Table S2. Analysis of variance for phenotypic traits of plant height.

Population	Week	Mean	SD	Range	CV	Kurtosis	Skewness
H_1	1	0.36	0.10	0.51	0.28	-0.14	0.26
	2	0.47	0.07	0.39	0.15	0.95	0.87
	3	0.99	1.04	6.80	1.05	15.25	3.55
	4	3.56	3.23	14.78	0.91	1.15	1.31
	5	8.60	5.29	21.59	0.62	-0.55	0.52
	6	14.48	5.72	25.06	0.39	-0.56	0.10
	7	16.46	5.71	25.51	0.35	-0.49	0.13
	8	22.51	6.08	23.12	0.27	-1.10	0.01
L_1	1	0.34	0.06	0.28	0.17	0.46	0.58
	2	0.42	0.05	0.27	0.12	0.00	-0.02
	3	0.57	0.17	1.53	0.30	44.95	6.08
	4	1.88	1.43	8.54	0.76	7.84	2.51
	5	4.64	3.78	14.17	0.81	-0.10	1.04
	6	8.35	5.64	20.01	0.68	-0.79	0.60
	7	10.33	5.66	20.63	0.55	-0.77	0.60
	8	15.95	7.10	26.97	0.44	-0.86	0.02
H_1H_2	1	0.50	0.05	0.24	0.10	-0.53	-0.38
	2	1.33	0.15	0.72	0.11	-0.15	0.08
	3	4.77	0.76	3.62	0.16	-0.48	0.01
	4	11.08	1.68	7.67	0.15	-0.39	0.38
	5	17.85	2.57	14.06	0.14	0.31	0.20
	6	23.86	2.86	14.11	0.12	-0.03	0.44
	7	27.79	2.65	13.13	0.10	-0.06	0.38
	8	32.33	2.90	11.42	0.09	-1.04	0.03
H_1L_2	1	0.47	0.04	0.19	0.09	-0.55	0.15
	2	1.24	0.13	0.64	0.11	-0.39	-0.07
	3	4.41	0.73	3.32	0.17	-0.63	-0.11
	4	10.23	1.66	8.61	0.16	0.05	0.00
	5	16.79	2.63	12.12	0.16	0.12	-0.42
	6	22.06	2.52	14.21	0.11	0.71	-0.60
	7	26.33	2.41	11.99	0.09	-0.18	-0.20
	8	30.30	2.51	11.93	0.08	-0.27	-0.31
L_1H_2	1	0.50	0.04	0.21	0.08	-0.23	0.07
	2	1.34	0.15	1.02	0.11	4.96	1.30
	3	4.69	0.78	3.97	0.17	0.24	0.23
	4	11.09	1.68	8.54	0.15	-0.09	-0.02
	5	18.31	2.37	10.96	0.13	-0.58	-0.18
	6	24.11	2.55	11.95	0.11	-0.22	0.06

	7	28.22	2.49	11.88	0.09	-0.37	-0.02
	8	32.52	2.67	14.75	0.08	0.54	-0.17
L_1L_2	1	0.28	0.03	0.11	0.10	-0.71	0.35
	2	0.72	0.08	0.47	0.11	0.66	0.15
	3	2.64	0.46	2.54	0.17	0.02	-0.01
	4	6.14	0.83	3.58	0.13	-0.42	-0.36
	5	9.87	1.23	6.19	0.12	-0.20	0.05
	6	13.04	1.61	8.53	0.12	-0.03	0.02
	7	15.20	1.56	7.32	0.10	-0.45	0.05
	8	17.45	1.71	8.09	0.10	-0.63	-0.15

Table S3. Significant loci of leaf number traits of *Arabidopsis thaliana* in H_1 generation.

Gene ID	Position	Chr	Alle	LR	Variation type	Gene Description
AT1G04390	1179676	1	A/A	27.578	3_prime_UTR	BTB/POZ domain-containing protein
AT3G51915	19271435	3	T/T	23.71	upstream_gene	hypothetical protein
AT3G52490	19459761	3	G/G	25.752	upstream_gene	Double Clp-N motif-containing P-loop nucleoside triphosphate hydrolases superfamily protein
AT3G52540	19493186	3	C/C	22.607	upstream_gene	ovate family protein 18
AT3G52680	19526403	3	T/T	24.427	upstream_gene	F-box/RNI-like/FBD-like domains-containing protein
AT3G52720	19545565	3	T/T	22.794	upstream_gene	alpha carbonic anhydrase 1
AT3G52820	19574638	3	A/A	22.884	synonymous	purple acid phosphatase 22
AT3G52940	19633275	3	A/A	22.834	5_prime_UTR	Ergosterol biosynthesis ERG4/ERG24 family
AT3G53040	19671189	3	A/A	27.934	upstream_gene	late embryogenesis abundant protein, putative / LEA protein
AT3G53100	19685456	3	C/C	23.166	missense	GDSL-like Lipase/Acylhydrolase superfamily protein
AT3G53300	19761946	3	T/T	24.365	synonymous	cytochrome P450, family 71, subfamily B, polypeptide 31
AT3G53310	19767634	3	T/T	22.681	missense	AP2/B3-like transcriptional factor family protein
AT3G53350	19783788	3	C/C	22.805	upstream_gene	ROP interactive partner 4
AT3G53360	19785037	3	C/C	23.746	missense	Tetratricopeptide repeat (TPR)-like superfamily protein
AT3G53490	19832638	3	C/C	23.22	5_prime_UTR	valine-tRNA ligase
AT3G53520	19842602	3	C/C	23.987	synonymous	UDP-glucuronic acid decarboxylase 1
AT3G53510	19843209	3	T/T	23.473	upstream_gene	ABC-2 type transporter family protein
AT3G53570	19864194	3	G/G	22.524	5_prime_UTR	serine/threonine-protein kinase AFC1
AT3G53560	19865114	3	G/G	22.869	upstream_gene	Tetratricopeptide repeat (TPR)-like superfamily protein
AT3G53680	19898564	3	T/T	23.133	upstream_gene	Acyl-CoA N-acyltransferase with RING/FYVE/PHD-type zinc finger domain-containing protein
AT3G53690	19898796	3	G/G	23.705	3_prime_UTR	RING/U-box superfamily protein
AT3G53730	19912749	3	C/C	22.569	synonymous	Histone superfamily protein

AT3G53850	19953405	3	T/T	25.113	upstream_gene	Uncharacterized protein family (UPF0497)
AT3G53890	19956315	3	T/T	25.16	5_prime_UTR	Ribosomal protein S21e
AT3G54000	19997815	3	T/T	22.771	missense	TIP41-like protein
AT3G54350	20126888	3	G/G	22.669	upstream_gene	Forkhead-associated (FHA) domain-containing protein
AT3G54440	20157324	3	T/T	23.669	upstream_gene	glycoside hydrolase family 2 protein
AT3G54460	20166886	3	G/G	23.796	missense	SNF2 domain-containing protein / helicase domain-containing protein / F-box family protein
AT3G54570	20204109	3	G/G	24.369	upstream_gene	Plant calmodulin-binding protein-like protein
AT3G54660	20237723	3	A/A	22.513	upstream_gene	glutathione reductase
AT3G54680	20244799	3	C/C	24.024	missense	proteophosphoglycan-like protein
AT3G54750	20264755	3	T/T	24.209	3_prime_UTR	downstream neighbor of Son

Table S4. Significant loci of leaf number traits of *Arabidopsis thaliana* in L_1 generation.

Gene ID	Position	Chr	Alle	LR	Variation type	Gene Description
AT2G20180	8707965	2	G/G	15.762	upstream_gene	phytochrome interacting factor 3-like 5
AT2G21730	9278561	2	C/C	15.738	upstream_gene	cinnamyl alcohol dehydrogenase homolog 2
AT2G21790	9292105	2	T/T	15.829	upstream_gene	ribonucleotide reductase 1
AT2G21800	9299512	2	C/C	16.153	missense&splice_region	essential meiotic endonuclease 1A
AT2G21810	9300062	2	A/A	19.316	upstream_gene	Cysteine/Histidine-rich C1 domain family protein
AT2G21900	9338442	2	G/G	15.952	upstream_gene	WRKY DNA-binding protein 59
AT2G22380	9501629	2	G/G	16.570	upstream_gene	tRNA-Ile
AT2G22690	9654200	2	G/G	17.425	upstream_gene	zinc ion binding protein
AT2G22740	9669229	2	T/T	18.412	upstream_gene	SU(VAR)3-9 homolog 6
AT2G22955	9773904	2	T/T	15.931	upstream_gene	ncRNA
AT2G23160	9865968	2	T/T	16.825	downstream_gene	F-box family protein
AT2G23880	10174259	2	T/T	16.301	upstream_gene	pseudo
AT2G23920	10181184	2	T/T	16.357	synonymous	hypothetical protein
AT2G24050	10225771	2	G/G	16.183	missense	MIF4G domain-containing protein / MA3 domain-containing protein
AT2G24080	10240879	2	G/G	16.865	upstream_gene	F-box protein (DUF295)
AT2G24103	10245676	2	A/A	17.413	upstream_gene	ncRNA
AT2G24100	10246268	2	G/G	16.664	synonymous	ATP-dependent DNA helicase
AT2G24110	10252248	2	G/G	15.752	upstream_gene	pseudogene
AT2G24150	10265728	2	A/A	17.333	synonymous	heptahelical protein 3
AT2G24170	10281500	2	A/A	17.061	upstream_gene	Endomembrane protein 70 protein family
AT2G24180	10282252	2	T/T	17.122	synonymous	cytochrome p450 71b6

AT2G24240	10312826	2	C/C	17.405	downstream_gene	BTB/POZ domain with WD40/YVTN repeat-like protein
AT2G27220	11642958	2	C/C	16.044	upstream_gene	BEL1-like homeodomain 5
AT2G42620	17751249	2	A/A	16.511	upstream_gene	RNI-like superfamily protein
AT2G42870	17837651	2	T/T	15.864	upstream_gene	phy rapidly regulated 1
AT2G43050	17903218	2	G/G	16.799	synonymous	Plant invertase/pectin methylesterase inhibitor superfamily
AT2G44280	18308954	2	C/C	16.158	upstream_gene	Major facilitator superfamily protein
AT2G44370	18322597	2	G/G	15.769	stop_gained	Cysteine/Histidine-rich C1 domain family protein
AT2G44460	18346527	2	G/G	16.709	missense	beta glucosidase 28
AT2G44470	18351258	2	T/T	17.005	upstream_gene	beta glucosidase 29
AT2G44540	18388016	2	G/G	16.088	missense	glycosyl hydrolase 9B9
AT2G44570	18400064	2	C/C	16.196	upstream_gene	uncharacterized LOC105268553
AT2G44578	18405943	2	A/A	16.122	upstream_gene	RING/U-box superfamily protein
AT2G44590	18406050	2	G/G	18.107	synonymous	DYNAMIN-like 1D
AT2G44770	18460227	2	A/A	16.803	3_prime_UTR	ELMO/CED-12 family protein
AT2G44870	18503999	2	C/C	17.431	synonymous	replicase polyprotein 1ab protein
AT2G44890	18513838	2	A/A	17.131	upstream_gene	cytochrome P450, family 704, subfamily A, polypeptide 1
AT2G45126	18608755	2	T/T	16.130	upstream_gene	hypothetical protein

Table S5. Significant loci of leaf number traits of *Arabidopsis thaliana* in H_1H_2 generation.

Gene ID	Position	Chr	Alle	LR	Variation type	Gene Description
AT4G35370	16812606	4	A/A	25.861	upstream_gene	Transducin/WD40 repeat-like superfamily protein
AT4G35390	16824511	4	A/A	25.725	upstream_gene	AT-hook protein of GA feedback 1
AT4G35450	16840350	4	T/T	26.276	synonymous	ankyrin repeat-containing protein 2
AT4G35650	16910021	4	T/T	26.372	3_prime_UTR	isocitrate dehydrogenase III
AT4G35640	16912376	4	C/C	30.504	upstream_gene	serine acetyltransferase 3;2
AT4G35660	16912804	4	T/T	26.829	missense	selection/upkeep of intraepithelial T-cells protein, putative (DUF241)
AT4G35690	16922831	4	A/A	26.567	3_prime_UTR	hypothetical protein (DUF241)
AT4G35700	16923091	4	T/T	26.626	upstream_gene	zinc finger (C2H2 type) family protein
AT4G35710	16925933	4	G/G	26.258	synonymous	DUF241 domain protein, putative (DUF241)
AT4G35740	16936193	4	T/T	25.776	5_prime_UTR	DEAD/DEAH box RNA helicase family protein
AT4G35733	16939206	4	T/T	26.405	downstream_gene	F-box SKIP23-like protein (DUF295)
AT4G35750	16941937	4	A/A	31.975	upstream_gene	SEC14 cytosolic factor family protein / phosphoglyceride transfer family protein
AT4G35760	16949146	4	T/T	25.538	upstream_gene	NAD(P)H dehydrogenase (quinone)s
AT4G35800	16968582	4	T/T	25.681	upstream_gene	RNA polymerase II large subunit

AT4G35810	16973927	4	A/A	27.215	downstream_gene	2-oxoglutarate (2OG) and Fe(II)-dependent oxygenase superfamily protein
AT4G35820	16975582	4	T/T	28.657	downstream_gene	2-oxoglutarate (2OG) and Fe(II)-dependent oxygenase superfamily protein
AT4G35830	16977104	4	G/G	26.318	synonymous	aconitase 1
AT4G35840	16982215	4	C/C	30.423	synonymous	RING/U-box superfamily protein
AT4G35837	16984099	4	C/C	27.84	upstream_gene	hypothetical protein
AT4G35860	16989499	4	T/T	27.357	upstream_gene	GTP-binding 2
AT4G35880	16995034	4	A/A	26.631	missense	Eukaryotic aspartyl protease family protein
AT4G35890	16995463	4	C/C	26.812	upstream_gene	winged-helix DNA-binding transcription factor family protein
AT4G35900	17005228	4	G/G	26.378	missense	Basic-leucine zipper (bZIP) transcription factor family protein
AT4G35910	17010429	4	G/G	26.32	synonymous	Adenine nucleotide alpha hydrolases-like superfamily protein
AT4G35920	17013293	4	C/C	29.706	splice_region&synonymous	PLAC8 family protein
AT4G35950	17026971	4	A/A	26.197	upstream_gene	RAC-like 6
AT4G35980	17032032	4	T/T	27.247	upstream_gene	uncharacterized protein
AT4G35987	17035948	4	C/C	25.544	synonymous	S-adenosyl-L-methionine-dependent methyltransferases superfamily protein
AT4G36080	17065428	4	G/G	25.521	synonymous	phosphotransferases/inositol or phosphatidylinositol kinase
AT4G36090	17079871	4	T/T	27.538	synonymous	oxidoreductase, 2OG-Fe(II) oxygenase family protein
AT4G36120	17098217	4	C/C	26.612	upstream_gene	filament-like protein (DUF869)
AT4G36150	17105735	4	G/G	29.271	missense	Disease resistance protein (TIR-NBS-LRR class) family
AT4G36140	17109354	4	T/T	28.279	upstream_gene	disease resistance protein (TIR-NBS-LRR class)
AT4G36170	17113170	4	T/T	26.039	upstream_gene	uncharacterized protein
AT4G36160	17118705	4	T/T	26.779	upstream_gene	NAC domain containing protein 76
AT4G36197	17129638	4	T/T	25.7	upstream_gene	tRNA-Glu
AT4G36195	17132359	4	T/T	27.065	downstream_gene	Serine carboxypeptidase S28 family protein
AT4G36250	17151190	4	A/A	25.56	synonymous	aldehyde dehydrogenase 3F1
AT4G36245	17154469	4	G/G	26.45	downstream_gene	tRNA-Pro
AT4G36260	17158846	4	T/T	29.078	upstream_gene	Lateral root primordium (LRP) protein-like protein
AT4G36360	17183722	4	T/T	25.86	upstream_gene	beta-galactosidase 3
AT4G36590	17265371	4	T/T	26.861	upstream_gene	MADS-box transcription factor family protein
AT4G36630	17278586	4	T/T	26.111	upstream_gene	Vacuolar sorting protein 39
AT4G36650	17285093	4	T/T	27.387	synonymous	plant-specific TFIIB-related protein
AT4G36648	17286112	4	C/C	28.978	upstream_gene	ncRNA
AT4G36740	17319172	4	A/A	28.308	upstream_gene	homeobox protein 40
AT4G37640	17688360	4	G/G	25.971	upstream_gene	calcium ATPase 2
AT4G39160	18243504	4	A/A	26.744	downstream_gene	Homeodomain-like superfamily protein

Table S6. Significant loci of leaf number traits of *Arabidopsis thaliana* in H_1L_2 generation.

Gene ID	Position	Chr	Alle	LR	Variation type	Gene Description
AT2G01950	444030	2	T/T	16.137	synonymous	BRI1-like 2
AT2G14940	6435223	2	C/C	15.945	upstream_gene	pseudo

AT2G15530	6769797	2	C/C	16.857	upstream_gene	RING/U-box superfamily protein
AT2G16700	7250404	2	A/A	16.038	downstream_gene	actin depolymerizing factor 5
AT2G16870	7310377	2	T/T	16.386	synonymous	Disease resistance protein (TIR-NBS-LRR class) family
AT2G27520	11762957	2	T/T	16.937	synonymous	F-box and associated interaction domains-containing protein
AT2G27570	11775564	2	G/G	15.903	stop_gained	P-loop containing nucleoside triphosphate hydrolases superfamily protein
AT2G27580	11776708	2	G/G	16.455	synonymous	A20/AN1-like zinc finger family protein
AT2G27650	11801094	2	G/G	16.256	upstream_gene	Ubiquitin carboxyl-terminal hydrolase-related protein
AT2G27700	11818626	2	C/C	16.016	upstream_gene	eukaryotic translation initiation factor 2 family protein / eIF-2 family protein
AT2G27740	11821497	2	G/G	16.697	upstream_gene	RAB6-interacting golgin (DUF662)
AT2G27880	11879001	2	A/A	15.97	downstream_gene	Argonaute family protein
AT2G27950	11905121	2	G/G	17.48	upstream_gene	Ring/U-Box superfamily protein
AT2G28060	11952520	2	T/T	16.746	upstream_gene	5'-AMP-activated protein kinase beta-2 subunit protein
AT2G28070	11958728	2	C/C	17.477	synonymous	ABC-2 type transporter family protein
AT2G28090	11977500	2	T/T	16.302	upstream_gene	Heavy metal transport/detoxification superfamily protein
AT2G28180	12017798	2	C/C	16.562	upstream_gene	cation/hydrogen exchanger family protein
AT4G18970	10383842	4	G/G	17.973	downstream_gene	GDSL-like Lipase/Acylhydrolase superfamily protein
AT4G19030	10425222	4	C/C	16.752	upstream_gene	NOD26-like major intrinsic protein 1
AT4G19038	10434247	4	T/T	16.769	upstream_gene	low-molecular-weight cysteine-rich 15
AT4G19080	10454395	4	A/A	15.909	upstream_gene	hypothetical protein (DUF594)
AT4G19180	10486438	4	G/G	16.468	synonymous	GDA1/CD39 nucleoside phosphatase family protein
AT4G19230	10522500	4	C/C	18.752	synonymous	cytochrome P450, family 707, subfamily A, polypeptide 1
AT4G19420	10594247	4	G/G	16.816	upstream_gene	Pectinacetylesterase family protein
AT4G19510	10637773	4	T/T	17.116	missense	Disease resistance protein (TIR-NBS-LRR class)
AT4G19570	10666275	4	G/G	16.374	missense	Chaperone DnaJ-domain superfamily protein
AT4G19590	10669551	4	T/T	17.418	upstream_gene	Chaperone DnaJ-domain superfamily protein
AT4G19690	10712682	4	A/A	16.241	downstream_gene	iron-regulated transporter 1
AT4G19710	10730964	4	T/T	18.143	downstream_gene	aspartate kinase-homoserine dehydrogenase ii
AT4G19720	10732207	4	A/A	17.801	upstream_gene	Glycosyl hydrolase family protein with chitinase insertion domain-containing protein
AT4G19990	10835039	4	C/C	16.863	splice_region&intron	FAR1-related sequence 1
AT4G22080	11704977	4	A/A	16.273	upstream_gene	root hair specific 14
AT4G22310	11792774	4	T/T	16.111	3_prime_UTR	Uncharacterized protein family (UPF0041)
AT4G22460	11839461	4	C/C	17.995	missense	Bifunctional inhibitor/lipid-transfer protein/seed storage 2S albumin superfamily protein
AT4G22770	11963882	4	C/C	16.098	synonymous	AT hook motif DNA-binding family protein

AT4G22980	12042678	4	C/C	16.057	downstream_gene	molybdenum cofactor sulfurase-like protein
AT4G23040	12077169	4	G/G	15.842	synonymous	Ubiquitin-like superfamily protein
AT4G23060	12091729	4	G/G	16.621	downstream_gene	IQ-domain 22
AT4G23250	12162949	4	T/T	17.906	missense	cysteine-rich receptor-like protein kinase 17
AT4G23370	12208086	4	A/A	17.054	synonymous	carboxyl-terminal peptidase

Table S7. Significant loci of leaf number traits of *Arabidopsis thaliana* in L_1H_2 generation.

Gene ID	Position	Chr	Alle	LR	Variation type	Gene Description
AT1G50030	18522648	1	T/T	16.144	splice_region&intron	target of rapamycin
AT2G14050	5909366	2	A/A	14.988	missense	minichromosome maintenance 9
AT2G14080	5923154	2	G/G	16.621	upstream_gene	Disease resistance protein (TIR-NBS-LRR class) family
AT2G14210	6018220	2	T/T	18.32	upstream_gene	AGAMOUS-like 44
AT2G14230	6033667	2	G/G	16.59	upstream_gene	pseudo
AT2G14245	6036670	2	G/G	16.15	upstream_gene	pseudo
AT2G14260	6042429	2	G/G	15.576	synonymous	proline iminopeptidase
AT2G14310	6070613	2	A/A	19.725	upstream_gene	pseudo
AT2G14378	6109824	2	C/C	17.262	upstream_gene	egg cell-secreted-like protein (DUF1278)
AT2G14380	6112001	2	T/T	15.217	upstream_gene	pseudo
AT2G14410	6126481	2	T/T	15.848	upstream_gene	pseudogene
AT2G14450	6146942	2	G/G	16.548	upstream_gene	pseudo
AT2G14470	6153982	2	G/G	17.612	upstream_gene	pseudo
AT2G14460	6157729	2	C/C	15.117	upstream_gene	uncharacterized protein
AT2G14510	6173991	2	G/G	16.712	missense	Leucine-rich repeat protein kinase family protein
AT2G14520	6188269	2	C/C	15.659	upstream_gene	CBS domain protein (DUF21)
AT2G14530	6188442	2	C/C	15.866	synonymous	TRICHOME BIREFRINGENCE-LIKE 13
AT2G14535	6189722	2	G/G	16.63	upstream_gene	pseudo
AT2G14550	6204446	2	G/G	15.042	intragenic	pseudogene
AT2G14560	6211175	2	T/T	15.454	upstream_gene	LURP-one-like protein (DUF567)
AT2G14610	6242269	2	C/C	17.068	missense	pathogenesis-related protein 1
AT2G14835	6372678	2	G/G	16.341	downstream_gene	RING/U-box superfamily protein
AT2G14870	6391425	2	C/C	14.805	missense	RNA-binding (RRM/RBD/RNP motifs) family protein
AT2G14960	6454752	2	A/A	15.653	upstream_gene	Auxin-responsive GH3 family protein
AT2G16000	6959493	2	G/G	16.251	downstream_gene	pseudo
AT2G16210	7032502	2	T/T	14.693	upstream_gene	Transcriptional factor B3 family protein
AT2G37550	15757207	2	G/G	15.016	missense	ARF-GAP domain 7
AT2G37600	15775643	2	A/A	14.875	upstream_gene	Ribosomal protein L36e family protein

AT2G37920	15872631	2	C/C	16.02	upstream_gene	copper ion transmembrane transporter
AT2G37930	15877470	2	G/G	15.304	downstream_gene	hypothetical protein (DUF3527)
AT2G38140	15976137	2	T/T	14.659	upstream_gene	plastid-specific ribosomal protein 4
AT2G38580	16137707	2	A/A	14.704	upstream_gene	Mitochondrial ATP synthase D chain-related protein
AT4G30840	15023742	4	T/T	15.243	upstream_gene	Transducin/WD40 repeat-like superfamily protein
AT4G33160	15994976	4	C/C	14.875	missense	F-box family protein
AT4G34170	16368244	4	A/A	17.871	missense	Galactose oxidase/kelch repeat superfamily protein
AT4G34220	16388827	4	A/A	16.641	upstream_gene	Leucine-rich repeat protein kinase family protein
AT4G34250	16396224	4	A/A	17.185	synonymous	3-ketoacyl-CoA synthase 16
AT4G35165	16739054	4	C/C	15.562	downstream_gene	egg cell-secreted-like protein (DUF1278)

Table S8. Significant loci of leaf number traits of *Arabidopsis thaliana* in L_1L_2 generation.

Gene ID	Position	Chr	Alle	LR	Variation type	Gene Description
AT1G15610	5371155	1	G/G	13.804	missense	uncharacterized protein
AT1G16440	5616819	1	G/G	14.19	missense	root hair specific 3
AT1G16670	5694727	1	G/G	15.542	upstream_gene	Protein kinase superfamily protein
AT2G18640	8086595	2	T/T	14.26	upstream_gene	geranylgeranyl pyrophosphate synthase 4
AT2G23310	9918041	2	C/C	14.193	synonymous	Rer1 family protein
AT2G24560	10435651	2	T/T	15.876	downstream_gene	GDSL-like Lipase/Acylhydrolase family protein
AT4G08540	5434413	4	T/T	15.51	intron	DNA-directed RNA polymerase II protein
AT4G08550	5440224	4	G/G	20.165	upstream_gene	electron carrier/ protein disulfide oxidoreductase
AT4G08596	5480616	4	G/G	16.827	upstream_gene	pseudo
AT4G08760	5595509	4	G/G	16.517	upstream_gene	uncharacterized protein
AT4G08930	5727717	4	G/G	14.725	upstream_gene	APR-like 6
AT4G08967	5755885	4	C/C	16.474	upstream_gene	hypothetical protein?
AT4G09012	5783346	4	C/C	14.043	synonymous	Mitochondrial ribosomal protein L27
AT4G09010	5784240	4	G/G	17.06	upstream_gene	ascorbate peroxidase 4
AT4G09020	5788426	4	G/G	13.86	missense	isoamylase 3
AT4G09040	5794568	4	A/A	14.493	3_prime_UTR	RNA-binding (RRM/RBD/RNP motifs) family protein
AT4G09110	5813385	4	G/G	14.022	missense	RING/U-box superfamily protein
AT4G09120	5815031	4	T/T	16.347	missense	RING/U-box superfamily protein
AT4G09140	5820656	4	C/C	14.815	missense	MUTL-homologue 1

AT4G09340	5931215	4	A/A	14.638	upstream_gene	SPla/RYanodine receptor (SPRY) domain-containing protein
AT4G09360	5940021	4	A/A	13.77	upstream_gene	NB-ARC domain-containing disease resistance protein
AT4G09450	5981989	4	G/G	13.986	upstream_gene	Duplicated homeodomain-like superfamily protein
AT4G09565	6043524	4	A/A	17.341	upstream_gene	pseudo
AT4G09900	6223885	4	C/C	13.768	missense	methyl esterase 12
AT4G09940	6235554	4	C/C	14.202	downstream_gene	P-loop containing nucleoside triphosphate hydrolases superfamily protein
AT4G10010	6269074	4	G/G	17.239	upstream_gene	Protein kinase superfamily protein
AT4G10110	6312643	4	A/A	13.891	upstream_gene	RNA-binding (RRM/RBD/RNP motifs) family protein
AT4G10130	6322790	4	T/T	15.057	upstream_gene	DNAJ heat shock N-terminal domain-containing protein
AT4G10160	6333072	4	G/G	15.939	upstream_gene	RING/U-box superfamily protein
AT4G10470	6476084	4	A/A	14.065	5_prime_UTR	uncharacterized protein
AT4G10520	6496066	4	G/G	15.027	upstream_gene	Subtilase family protein
AT4G10510	6503458	4	G/G	14.949	downstream_gene	Subtilase family protein
AT4G10530	6509919	4	C/C	15.567	synonymous	Subtilase family protein
AT4G10540	6516108	4	C/C	14.868	upstream_gene	Subtilase family protein
AT4G14320	8242022	4	A/A	15.283	stop_gained	Zinc-binding ribosomal protein family protein

Table S9. Significant loci of plant height traits of *Arabidopsis thaliana* in H_1 generation.

Gene ID	Position	Chr	Alle	LR	Variation type	Gene Description
AT1G01540	198466	1	A/A	89.117	3_prime_UTR	Protein kinase superfamily protein
AT1G01660	242603	1	C/C	95.911	synonymous	RING/U-box superfamily protein
AT1G01700	264633	1	A/A	89.591	upstream_gene	RHO guanyl-nucleotide exchange factor 2
AT1G01720	267588	1	C/C	90.46	upstream_gene	NAC (No Apical Meristem) domain transcriptional regulator superfamily protein
AT1G01830	304984	1	A/A	92.95	upstream_gene	ARM repeat superfamily protein
AT1G17840	6143631	1	T/T	89.826	synonymous	white-brown complex-like protein
AT1G17850	6144071	1	T/T	88.653	downstream_gene	Rhodanese/Cell cycle control phosphatase superfamily protein
AT1G17870	6151973	1	T/T	90.267	missense	ethylene-dependent gravitropism-deficient and yellow-green-like 3
AT1G17890	6155586	1	T/T	87.717	missense	NAD(P)-binding Rossmann-fold superfamily protein
AT1G17910	6159248	1	C/C	88.672	synonymous	Wall-associated kinase family protein
AT1G18415	6343945	1	A/A	88.305	splice_region&intron	ncRNA
AT1G18810	6488742	1	A/A	102.872	upstream_gene	phytochrome kinase substrate-like protein
AT1G19110	6601939	1	C/C	89.391	upstream_gene	inter-alpha-trypsin inhibitor heavy chain-like protein

AT1G19310	6678575	1	C/C	103.573	upstream_gene	RING/U-box superfamily protein
AT1G19340	6691997	1	C/C	94.314	downstream_gene	Methyltransferase MT-A70 family protein
AT1G19390	6703658	1	T/T	88.361	upstream_gene	Wall-associated kinase family protein
AT1G19396	6710274	1	A/A	92.787	3_prime_UTR	uncharacterized protein
AT1G19397	6711601	1	T/T	95.648	splice_region&intron	uncharacterized protein
AT1G19400	6714461	1	A/A	93.964	upstream_gene	Erythronate-4-phosphate dehydrogenase family protein
AT1G19415	6723866	1	T/T	88.555	downstream_gene	pseudo
AT1G19460	6733505	1	C/C	96.77	upstream_gene	Galactose oxidase/kelch repeat superfamily protein
AT1G19470	6740532	1	T/T	87.365	upstream_gene	Galactose oxidase/kelch repeat superfamily protein
AT1G19480	6747708	1	T/T	103.251	downstream_gene	DNA glycosylase superfamily protein
AT1G19490	6752016	1	T/T	89.964	synonymous	Basic-leucine zipper (bZIP) transcription factor family protein
AT1G19620	6781335	1	A/A	88.371	upstream_gene	uncharacterized protein
AT1G19670	6808235	1	C/C	98.005	upstream_gene	chlorophyllase 1
AT1G19715	6819188	1	T/T	87.806	missense	chlorophyllase 1
AT1G19770	6833252	1	C/C	111.343	synonymous	Mannose-binding lectin superfamily protein
AT1G19835	6856106	1	C/C	96.86	3_prime_UTR	filament-like protein (DUF869)
AT1G19960	6927201	1	G/G	96.982	upstream_gene	transcription factor
AT1G20000	6942109	1	C/C	103.818	upstream_gene	TBP-associated factor 11B
AT1G20110	6967455	1	T/T	105.533	upstream_gene	RING/FYVE/PHD zinc finger superfamily protein
AT1G20140	6987043	1	G/G	91.075	synonymous	SKP1-like 4
AT1G20160	6992365	1	C/C	101.859	missense	Subtilisin-like serine endopeptidase family protein
AT1G20320	7033779	1	A/A	92.18	3_prime_UTR	Haloacid dehalogenase-like hydrolase (HAD) superfamily protein
AT1G20470	7094842	1	C/C	87.624	3_prime_UTR	SAUR-like auxin-responsive protein family
AT1G20480	7094908	1	A/A	97.159	3_prime_UTR	AMP-dependent synthetase and ligase family protein
AT1G20500	7101004	1	T/T	87.967	synonymous	AMP-dependent synthetase and ligase family protein
AT1G20640	7157497	1	T/T	93.376	synonymous	Plant regulator RWP-RK family protein
AT1G20650	7161985	1	T/T	90.504	upstream_gene	Protein kinase superfamily protein
AT1G20670	7172153	1	C/C	105.512	upstream_gene	DNA-binding bromodomain-containing protein
AT1G20735	7201332	1	T/T	108.356	downstream_gene	F-box and associated interaction domains-containing protein
AT1G20770	7218980	1	C/C	103.883	upstream_gene	coiled-coil protein
AT1G20795	7227405	1	A/A	97.046	synonymous	F-box family protein
AT1G20960	7302894	1	A/A	90.38	synonymous	U5 small nuclear ribonucleoprotein helicase
AT1G21000	7338733	1	A/A	106.414	intron	PLATZ transcription factor family protein
AT1G21060	7368659	1	C/C	92.556	upstream_gene	Serine/Threonine-kinase, putative (Protein of unknown function, DUF547)
AT1G21100	7392007	1	T/T	89.998	upstream_gene	O-methyltransferase family protein
AT1G21130	7400458	1	G/G	87.322	missense	O-methyltransferase family protein
AT1G21250	7441704	1	G/G	87.562	missense	cell wall-associated kinase
AT1G21270	7442874	1	G/G	90.74	upstream_gene	wall-associated kinase 2
AT1G21323	7463401	1	A/A	92.554	upstream_gene	dual specificity kinase
AT1G21320	7464939	1	G/G	99.161	missense	nucleic acid/nucleotide binding protein
AT1G21395	7492165	1	G/G	87.699	missense	uncharacterized protein

AT1G21480	7519823	1	T/T	102.774	splice_region&intron	Exostosin family protein
AT1G21475	7527818	1	C/C	107.073	upstream_gene	hypothetical protein (DUF506)
AT1G21550	7553666	1	C/C	89.544	missense	Calcium-binding EF-hand family protein
AT1G21730	7635887	1	T/T	89.957	synonymous	P-loop containing nucleoside triphosphate hydrolases superfamily protein
AT1G21740	7641926	1	A/A	93.547	missense	DUF630 family protein, putative (DUF630 and DUF632)
AT1G21738	7643594	1	T/T	99.096	upstream_gene	uncharacterized protein
AT1G21750	7645823	1	G/G	96.019	synonymous	PDI-like 1-1
AT1G21760	7648294	1	C/C	88.211	upstream_gene	F-box protein 7
AT1G21800	7659225	1	G/G	102.959	upstream_gene	tRNA-Gln
AT1G22830	8073037	1	A/A	88.551	upstream_gene	Tetratricopeptide repeat (TPR)-like superfamily protein
AT1G22850	8081729	1	A/A	102.911	synonymous	SNARE associated Golgi protein family
AT1G23000	8143262	1	T/T	96.166	upstream_gene	Heavy metal transport/detoxification superfamily protein
AT1G23010	8147348	1	C/C	92.892	5_prime_UTR	Cupredoxin superfamily protein

Table S10. Significant loci of plant height traits of *Arabidopsis thaliana* in L_1 generation.

Gene ID	Position	Chr	Alle	LR	Variation type	Gene Description
AT1G01183	80452	1	T/T	42.324	upstream_gene	ncRNA
AT1G01190	83632	1	C/C	42.055	missense	cytochrome P450, family 78, subfamily A, polypeptide 8
AT1G01225	96024	1	T/T	58.279	5_prime_UTR	NC domain-containing protein-like protein
AT1G01650	237838	1	G/G	42.904	upstream_gene	SIGNAL PEPTIDE PEPTIDASE-LIKE 4
AT1G01690	251010	1	G/G	47.026	missense	putative recombination initiation defects 3
AT1G01760	278383	1	C/C	39.834	synonymous	denosine deaminases acting on tRNA
AT1G02074	368476	1	A/A	38.911	upstream_gene	hypothetical protein
AT1G02190	419964	1	A/A	41.594	downstream_gene	Fatty acid hydroxylase superfamily
AT1G02310	462817	1	G/G	39.658	upstream_gene	Glycosyl hydrolase superfamily protein
AT1G02470	518006	1	C/C	40.685	upstream_gene	Polyketide cyclase/dehydrase and lipid transport superfamily protein
AT1G02710	594568	1	C/C	40.898	upstream_gene	glycine-rich protein

AT1G02750	604011	1	A/A	40.029	synonymous	Drought-responsive family protein
AT1G02940	663822	1	C/C	38.771	missense	glutathione S-transferase (class phi) 5
AT1G03060	731147	1	T/T	45.32	upstream_gene	BEACH-DOMAIN HOMOLOG A1
AT1G03080	737340	1	G/G	40.536	5_prime_UTR	kinase interacting (KIP1-like) family protein
AT1G03130	757790	1	C/C	42.615	upstream_gene	photosystem I subunit D-2
AT1G04400	1192200	1	T/T	42.981	upstream_gene	cryptochrome 2
AT1G04540	1238774	1	T/T	41.013	missense	Calcium-dependent lipid-binding (CaLB domain) family protein
AT1G04570	1253168	1	C/C	40.155	upstream_gene	Major facilitator superfamily protein
AT1G05870	1772444	1	A/A	39.548	3_prime_UTR	hypothetical protein (DUF1685)
AT1G06840	2107455	1	T/T	38.469	upstream_gene	Leucine-rich repeat protein kinase family protein
AT1G12120	4120891	1	G/G	40.156	upstream_gene	hypothetical protein (DUF863)
AT1G13000	4443986	1	A/A	47.376	upstream_gene	transmembrane protein, putative (DUF707)
AT1G17750	6108188	1	T/T	39.161	synonymous	PEP1 receptor 2
AT1G19480	6750567	1	T/T	40.221	downstream_gene	DNA glycosylase superfamily protein
AT1G20130	6977764	1	A/A	41.092	5_prime_UTR	GDSL-like Lipase/Acylhydrolase superfamily protein
AT1G58370	21683143	1	G/G	39.711	upstream_gene	glycosyl hydrolase family 10 protein / carbohydrate-binding domain-containing protein
AT1G72720	27379249	1	A/A	40.935	upstream_gene	hypothetical protein (DUF3511)
AT2G01940	436686	2	G/G	43.882	downstream_gene	C2H2-like zinc finger protein
AT2G03460	1043923	2	T/T	40.269	missense	Galactose oxidase/kelch repeat superfamily protein
AT3G05510	1595362	3	C/C	43.366	upstream_gene	Phospholipid/glycerol acyltransferase family protein
AT5G18580	6178166	5	G/G	38.819	synonymous	tonneau 2 (TON2)
AT5G18620	6204803	5	A/A	45.378	upstream_gene	chromatin remodeling factor17
AT5G18760	6259341	5	C/C	43.089	stop_gained	RING/U-box superfamily protein
AT5G18770	6261630	5	G/G	39.076	missense	F-box/FBD-like domains containing protein
AT5G18980	6337940	5	C/C	45.769	missense	ARM repeat superfamily protein
AT5G19010	6347976	5	G/G	42.395	5_prime_UTR	mitogen-activated protein kinase 16
AT5G19090	6383673	5	A/A	44.537	upstream_gene	Heavy metal transport/detoxification superfamily protein
AT5G19210	6463757	5	T/T	38.633	3_prime_UTR	P-loop containing nucleoside triphosphate hydrolases superfamily protein
AT5G19290	6492904	5	T/T	52.994	upstream_gene	alpha/beta-Hydrolases superfamily protein
AT5G19420	6555945	5	C/C	41.587	upstream_gene	Regulator of chromosome condensation (RCC1) family with FYVE zinc finger domain-containing protein
AT5G19660	6643609	5	G/G	46.337	synonymous	SITE-1 protease
AT5G19690	6656521	5	T/T	45.118	missense	staurosporin and temperature sensitive 3-like A
AT5G20060	6778999	5	G/G	43.691	synonymous	alpha/beta-Hydrolases superfamily protein
AT5G20250	6833250	5	C/C	54.178	upstream_gene	Raffinose synthase family protein
AT5G20270	6847539	5	A/A	39.542	upstream_gene	heptahelical transmembrane protein1
AT5G20340	6879026	5	C/C	44.834	downstream_gene	beta-1,3-glucanase 5
AT5G20360	6886926	5	G/G	38.802	upstream_gene	Octicosapeptide/Phox/Bem1p (PB1) domain-containing protein / tetratricopeptide repeat (TPR)-containing protein
AT5G20550	6953472	5	G/G	43.311	synonymous	2-oxoglutarate (2OG) and Fe(II)-dependent oxygenase superfamily protein
AT5G20560	6953543	5	A/A	48.899	upstream_gene	Glycosyl hydrolase superfamily protein
AT5G20690	7000696	5	C/C	40.446	upstream_gene	Leucine-rich repeat protein kinase family protein

AT5G20830	7052627	5	T/T	40.488	missense	sucrose synthase 1
AT5G21040	7147027	5	T/T	39.233	5_prime_UTR	F-box protein 2
AT5G21140	7192173	5	A/A	38.54	upstream_gene	embryo defective 1379
AT5G22040	7297366	5	G/G	38.909	synonymous	ubiquitin carboxyl-terminal hydrolase
AT5G22070	7308807	5	A/A	40.201	missense	Core-2/I-branching beta-1,6-N-acetylglucosaminyltransferase family protein
AT5G22110	7334253	5	C/C	43.953	upstream_gene	DNA polymerase epsilon subunit B2
AT5G22130	7337646	5	T/T	43.157	missense	mannosyltransferase family protein
AT5G22140	7340509	5	G/G	38.644	synonymous	FAD/NAD(P)-binding oxidoreductase family protein
AT5G22180	7355641	5	C/C	38.872	upstream_gene	uncharacterized protein
AT5G22270	7378043	5	A/A	45.431	upstream_gene	uncharacterized protein
AT5G23040	7729889	5	A/A	44.688	synonymous	cell growth defect factor-like protein (DUF3353)
AT5G23110	7755771	5	C/C	45.455	upstream_gene	cell growth defect factor-like protein (DUF3353)
AT5G23270	7840649	5	C/C	42.142	missense	sugar transporter 11
AT5G24510	8374778	5	A/A	42.87	upstream_gene	60S acidic ribosomal protein family
AT5G25130	8668812	5	A/A	40.819	missense	cytochrome P450, family 71, subfamily B, polypeptide 12
AT5G25265	8757448	5	G/G	39.735	upstream_gene	Hyp O-arabinosyltransferase-like protein
AT5G25300	8782679	5	T/T	41.533	missense	F-box protein
AT5G25570	8903038	5	C/C	41.481	3_prime_UTR	polyamine-modulated factor 1-binding protein
AT5G25900	9036763	5	G/G	39.23	synonymous	GA requiring 3
AT5G35420	13659074	5	C/C	46.576	upstream_gene	pseudo

Table S11. Significant loci of plant height traits of *Arabidopsis thaliana* in H_1H_2 generation.

Gene ID	Position	Chr	Alle	LR	Variation type	Gene Description
AT4G35640	16911160	4	A/A	34.44	upstream_gene	serine acetyltransferase 3;2
AT4G35660	16912550	4	A/A	31.517	5_prime_UTR	selection/upkeep of intraepithelial T-cells protein, putative (DUF241)

AT4G35770	16941163	4	A/A	32.934	upstream_gene	Rhodanese/Cell cycle control phosphatase superfamily protein
AT4G35840	16982158	4	T/T	33.228	synonymous	RING/U-box superfamily protein
AT4G35837	16984099	4	C/C	31.736	upstream_gene	hypothetical protein
AT4G35860	16989499	4	T/T	31.511	upstream_gene	GTP-binding 2
AT4G35890	16995463	4	C/C	32.942	upstream_gene	winged-helix DNA-binding transcription factor family protein
AT4G35900	17001820	4	A/A	31.752	upstream_gene	Basic-leucine zipper (bZIP) transcription factor family protein
AT4G35910	17010429	4	G/G	31.928	synonymous	Adenine nucleotide alpha hydrolases-like superfamily protein
AT4G35920	17013293	4	C/C	37.245	splice_region&synonymous	PLAC8 family protein
AT4G35950	17026971	4	A/A	31.862	upstream_gene	RAC-like 6
AT4G35980	17032032	4	T/T	34.099	upstream_gene	uncharacterized protein
AT4G35985	17038768	4	G/G	32.393	upstream_gene	Senescence/dehydration-associated protein-like protein
AT4G36080	17072182	4	A/A	32.728	intron	phosphotransferases/inositol or phosphatidylinositol kinase
AT4G36090	17076111	4	G/G	31.565	downstream_gene	oxidoreductase, 2OG-Fe(II) oxygenase family protein
AT4G36120	17098217	4	C/C	32.843	upstream_gene	filament-like protein (DUF869)
AT4G36130	17098479	4	T/T	32.35	synonymous	Ribosomal protein L2 family
AT4G36140	17106980	4	T/T	34.742	upstream_gene	disease resistance protein (TIR-NBS-LRR class)
AT4G36150	17107236	4	C/C	36.209	missense	Disease resistance protein (TIR-NBS-LRR class) family
AT4G36160	17118705	4	T/T	33.023	upstream_gene	NAC domain containing protein 76
AT4G36197	17129567	4	T/T	31.35	upstream_gene	tRNA-Glu
AT4G36250	17152127	4	T/T	32.406	splice_region&intron	aldehyde dehydrogenase 3F1
AT4G36350	17180090	4	C/C	33.309	upstream_gene	purple acid phosphatase 25
AT4G36360	17180381	4	G/G	32.827	splice_region&intron	beta-galactosidase 3
AT4G36380	17188557	4	A/A	33.908	missense	Cytochrome P450 superfamily protein
AT4G36520	17238346	4	T/T	31.349	upstream_gene	Chaperone DnaJ-domain superfamily protein
AT4G36530	17240199	4	G/G	31.35	missense	alpha/beta-Hydrolases superfamily protein
AT4G36590	17265352	4	C/C	32.518	upstream_gene	MADS-box transcription factor family protein
AT4G36630	17273803	4	C/C	34.772	synonymous	Vacuolar sorting protein 39
AT4G36650	17285093	4	T/T	34.458	synonymous	plant-specific TFIIB-related protein
AT4G36648	17286102	4	G/G	34.577	upstream_gene	ncRNA
AT4G36670	17288961	4	G/G	32.485	synonymous	Major facilitator superfamily protein
AT4G36680	17297373	4	G/G	34.559	upstream_gene	Tetratricopeptide repeat (TPR)-like superfamily protein
AT4G37640	17688360	4	G/G	32.975	upstream_gene	calcium ATPase 2
AT4G37870	17804979	4	A/A	31.414	splice_region&intron	phosphoenolpyruvate carboxykinase 1
AT4G38260	17941125	4	A/A	33.598	upstream_gene	transport/golgi organization-like protein (DUF833)

Table S12. Significant loci of plant height traits of *Arabidopsis thaliana* in H_1L_2 generation.

Gene ID	Position	Chr	Alle	LR	Variation type	Gene Description
AT2G26750	11399857	2	C/C	29.725	upstream_gene	alpha/beta-Hydrolases superfamily protein
AT2G27520	11762957	2	T/T	36.851	synonymous	F-box and associated interaction domains-containing protein
AT2G27530	11767598	2	C/C	31.203	upstream_gene	Ribosomal protein L1p/L10e family
AT2G27550	11771645	2	T/T	30.95	upstream_gene	centroradiali
AT2G27570	11775564	2	G/G	29.982	stop_gained	P-loop containing nucleoside triphosphate hydrolases superfamily protein
AT2G27580	11777295	2	A/A	31.06	5_prime_UTR	A20/AN1-like zinc finger family protein
AT2G27560	11778150	2	T/T	31.835	upstream_gene	tRNA-Lys
AT2G27600	11783027	2	T/T	29.645	splice_region&intron	AAA-type ATPase family protein
AT2G27630	11793303	2	G/G	29.759	upstream_gene	Ubiquitin carboxyl-terminal hydrolase-related protein
AT2G27650	11795740	2	G/G	31.427	missense	Ubiquitin carboxyl-terminal hydrolase-related protein
AT2G27680	11809302	2	A/A	30.706	upstream_gene	NAD(P)-linked oxidoreductase superfamily protein
AT2G27700	11819297	2	A/A	31.662	upstream_gene	eukaryotic translation initiation factor 2 family protein / eIF-2 family protein
AT2G27740	11821497	2	G/G	34.077	upstream_gene	RAB6-interacting golgin (DUF662)
AT2G27770	11836383	2	A/A	31.75	upstream_gene	DUF868 family protein (DUF868)
AT2G27800	11855269	2	T/T	30.559	upstream_gene	Tetratricopeptide repeat (TPR)-like superfamily protein
AT2G27830	11861220	2	T/T	32.803	missense	uncharacterized protein
AT2G27880	11878388	2	A/A	32.931	downstream_gene	Argonaute family protein
AT2G27920	11890137	2	T/T	29.978	upstream_gene	serine carboxypeptidase-like 51
AT2G27940	11897966	2	G/G	29.79	stop_gained	RING/U-box superfamily protein
AT2G27970	11911452	2	A/A	31.694	downstream_gene	CDK-subunit 2
AT2G28020	11929973	2	C/C	30.82	upstream_gene	mediator of RNA polymerase II transcription subunit
AT2G28030	11938195	2	G/G	31.628	upstream_gene	Eukaryotic aspartyl protease family protein
AT2G28040	11941982	2	A/A	31.356	upstream_gene	Eukaryotic aspartyl protease family protein
AT2G28060	11952520	2	T/T	30.735	upstream_gene	5'-AMP-activated protein kinase beta-2 subunit protein
AT2G28085	11970595	2	G/G	33.033	upstream_gene	SAUR-like auxin-responsive protein family
AT2G28140	11994680	2	C/C	31.901	upstream_gene	enabled-like protein (DUF1635)
AT2G28180	12017798	2	C/C	33.734	upstream_gene	cation/hydrogen exchanger family protein
AT2G28380	12136618	2	C/C	32.815	upstream_gene	dsRNA-binding protein 2
AT2G28405	12158988	2	G/G	30.802	upstream_gene	ow-molecular-weight cysteine-rich 32
AT2G28426	12159508	2	A/A	31.543	upstream_gene	uncharacterized protein
AT2G28430	12159565	2	A/A	33.378	3_prime_UTR	zinc finger, C3HC4 type family protein
AT2G28440	12161924	2	G/G	31.518	synonymous	proline-rich family protein
AT2G28470	12171313	2	C/C	30.611	missense	beta-galactosidase 8
AT2G28500	12189131	2	G/G	31.105	upstream_gene	LOB domain-containing protein 11
AT2G28671	12302033	2	A/A	31.578	3_prime_UTR	uncharacterized protein
AT2G28670	12302039	2	T/T	31.578	upstream_gene	Disease resistance-responsive (dirigent-like protein) family protein
AT2G28720	12325475	2	A/A	30.633	upstream_gene	Histone superfamily protein
AT2G28780	12341545	2	T/T	30.899	synonymous	P-hydroxybenzoic acid efflux pump subunit

Table S13. Significant loci of plant height traits of *Arabidopsis thaliana* in L_1H_2 generation.

Gene ID	Position	Chr	Alle	LR	Variation type	Gene Description
AT1G62305	23025948	1	A/A	16.694	downstream_gene	Core-2/I-branching beta-1,6-N-acetylglucosaminyltransferase family protein
AT1G62310	23035331	1	T/T	17.567	downstream_gene	transcription factor jumonji (jmjC) domain-containing protein
AT1G63180	23430391	1	A/A	17.584	upstream_gene	UDP-D-glucose/UDP-D-galactose 4-epimerase 3
AT2G14210	6018220	2	T/T	18.521	upstream_gene	AGAMOUS-like 44
AT2G14310	6070613	2	A/A	19.489	upstream_gene	pseudo
AT2G14370	6098717	2	T/T	17.503	downstream_gene	pseudo
AT3G02520	531906	3	A/A	16.368	upstream_gene	pseudo
AT4G29020	14301486	4	C/C	16.429	upstream_gene	glycine-rich protein
AT4G30810	15006104	4	A/A	16.373	3_prime_UTR	serine carboxypeptidase-like 29
AT4G30830	15019813	4	T/T	16.482	upstream_gene	myosin-like protein (Protein of unknown function, DUF593)
AT4G30840	15023742	4	T/T	16.116	upstream_gene	Transducin/WD40 repeat-like superfamily protein
AT4G30880	15030366	4	G/G	17.378	upstream_gene	Bifunctional inhibitor/lipid-transfer protein/seed storage 2S albumin superfamily protein
AT4G30960	15069872	4	C/C	16.119	downstream_gene	SOS3-interacting protein 3
AT4G31080	15120915	4	C/C	16.15	5_prime_UTR	vintegral membrane metal-binding family protein (DUF2296)
AT4G31130	15137686	4	A/A	16.612	synonymous	keratin-associated protein (DUF1218)
AT4G31170	15160211	4	G/G	16.523	upstream_gene	Protein kinase superfamily protein
AT4G31250	15185358	4	C/C	19.03	upstream_gene	Leucine-rich repeat protein kinase family protein
AT4G31354	15219261	4	G/G	16.764	missense	uncharacterized protein?
AT4G31390	15233791	4	G/G	18.02	synonymous	Protein kinase superfamily protein
AT4G31398	15242052	4	G/G	16.569	upstream_gene	ncRNA
AT4G31570	15301811	4	G/G	17.162	synonymous	nucleoporin
AT4G31580	15306761	4	C/C	19.448	5_prime_UTR	serine/arginine-rich 22
AT4G31630	15325840	4	A/A	17.862	synonymous	Transcriptional factor B3 family protein
AT4G31640	15334166	4	T/T	18.902	downstream_gene	transcriptional factor B3 family protein
AT4G31660	15334784	4	G/G	16.133	missense	AP2/B3-like transcriptional factor family protein
AT4G31650	15337116	4	G/G	17.286	downstream_gene	Transcriptional factor B3 family protein
AT4G31710	15355099	4	G/G	20.036	downstream_gene	glutamate receptor 2.4
AT4G31720	15359102	4	G/G	16.957	upstream_gene	TBP-associated factor II 15
AT4G31760	15372756	4	T/T	16.668	upstream_gene	peroxidase superfamily protein
AT4G31820	15395819	4	T/T	16.445	upstream_gene	Phototropic-responsive NPH3 family protein
AT4G31870	15410208	4	G/G	17.771	missense	glutathione peroxidase 7
AT4G32000	15475392	4	C/C	16.759	splice_region&intron	Protein kinase superfamily protein
AT4G32710	15783140	4	A/A	17.5	synonymous	Protein kinase superfamily protein
AT4G32750	15800182	4	T/T	16.264	upstream_gene	uncharacterized protein

AT4G33670	16175804	4	G/G	16.596	upstream_gene	NAD(P)-linked oxidoreductase superfamily protein
AT4G34230	16386814	4	A/A	17.285	3_prime_UTR	cinnamyl alcohol dehydrogenase 5
AT4G34220	16388827	4	A/A	17.799	upstream_gene	Leucine-rich repeat protein kinase family protein
AT4G34710	16561189	4	A/A	17.859	missense	arginine decarboxylase 2
AT4G34870	16615259	4	G/G	17.655	3_prime_UTR	rotamase cyclophilin 5
AT4G34980	16659152	4	G/G	17.005	synonymous	subtilisin-like serine protease 2
AT4G35170	16738169	4	T/T	17.015	missense	Late embryogenesis abundant (LEA) hydroxyproline-rich glycoprotein family
AT4G35165	16739054	4	C/C	17.661	downstream_gene	egg cell-secreted-like protein (DUF1278)
AT4G35260	16772543	4	C/C	16.291	downstream_gene	isocitrate dehydrogenase 1
AT4G35280	16790188	4	T/T	20.866	upstream_gene	C2H2-like zinc finger protein
AT4G35320	16807656	4	A/A	18.272	upstream_gene	uncharacterized protein
AT4G35370	16812597	4	A/A	17.367	upstream_gene	Transducin/WD40 repeat-like superfamily protein
AT4G35360	16818295	4	A/A	21.047	upstream_gene	pantothenate kinase
AT4G35380	16825777	4	A/A	19.761	synonymous	SEC7-like guanine nucleotide exchange family protein
AT4G35390	16829046	4	C/C	17.672	upstream_gene	AT-hook protein of GA feedback 1
AT4G35480	16854032	4	G/G	16.113	upstream_gene	RING-H2 finger A3B
AT4G35500	16858673	4	A/A	16.872	synonymous	Protein kinase superfamily protein
AT4G35510	16862441	4	G/G	16.522	upstream_gene	PHD finger-like protein
AT4G35520	16867793	4	C/C	16.502	missense	MUTL protein homolog 3
AT4G35540	16872155	4	A/A	19.396	upstream_gene	POLLEN-EXPRESSED TRANSCRIPTION FACTOR 2
AT4G35650	16910021	4	T/T	16.544	3_prime_UTR	isocitrate dehydrogenase III

Table S14. Significant loci of plant height traits of *Arabidopsis thaliana* in L_1L_2 generation.

Gene ID	Position	Chr	Alle	LR	Variation type	Gene Description
AT1G12770	4357726	1	T/T	13.844	downstream_gene	P-loop containing nucleoside triphosphate hydrolases superfamily protein
AT1G13130	4470004	1	A/A	16.073	upstream_gene	Cellulase (glycosyl hydrolase family 5) protein
AT1G13460	4621213	1	A/A	13.822	downstream_gene	Protein phosphatase 2A regulatory B subunit family protein
AT1G14510	4968885	1	A/A	15.33	upstream_gene	alfin-like 7
AT1G14670	5038292	1	C/C	15.238	synonymous	Endomembrane protein 70 protein family
AT1G14880	5136311	1	C/C	13.645	upstream_gene	PLANT CADMIUM RESISTANCE 1
AT1G15040	5183146	1	G/G	14.759	upstream_gene	Class I glutamine amidotransferase-like superfamily protein
AT1G15060	5186968	1	T/T	13.772	5_prime_UTR	alpha/beta hydrolase family protein
AT1G15610	5371155	1	G/G	14.352	missense	uncharacterized protein
AT1G15640	5379874	1	T/T	16.769	synonymous	uncharacterized protein

AT1G15750	5421052	1	T/T	13.846	upstream_gene	Transducin family protein / WD-40 repeat family protein
AT1G16440	5616819	1	G/G	14.745	missense	root hair specific 3
AT1G16670	5694727	1	G/G	15.786	upstream_gene	Protein kinase superfamily protein
AT2G18640	8086595	2	T/T	14.905	upstream_gene	geranylgeranyl pyrophosphate synthase 4
AT2G46420	19056541	2	G/G	14.351	splice_region&intron	helicase with zinc finger protein
AT2G46430	19059883	2	G/G	13.953	splice_region&intron	cyclic nucleotide gated channel 3
AT2G46440	19062038	2	T/T	13.85	5_prime_UTR	cyclic nucleotide-gated channels
AT2G46450	19067899	2	T/T	14.653	synonymous	cyclic nucleotide-gated channel 12
AT2G46455	19070476	2	C/C	14.195	missense	OxaA/YidC-like membrane insertion protein
AT2G46495	19089194	2	A/A	14.989	upstream_gene	RING/U-box superfamily protein
AT2G46662	19157957	2	T/T	14.214	missense	hypothetical protein
AT2G46700	19186626	2	A/A	16.143	upstream_gene	CDPK-related kinase 3
AT2G46800	19239249	2	G/G	13.651	missense	zinc transporter
AT2G46840	19250296	2	A/A	14.643	upstream_gene	hypothetical protein (DOMAIN OF UNKNOWN FUNCTION 724 4)
AT2G47060	19339424	2	T/T	16.6	upstream_gene	Protein kinase superfamily protein
AT2G47070	19339925	2	A/A	13.89	missense	squamosa promoter binding protein-like 1
AT2G47090	19342064	2	T/T	13.695	missense	zinc ion binding/nucleic acid binding protein
AT2G47420	19456710	2	A/A	13.898	upstream_gene	Ribosomal RNA adenine dimethylase family protein
AT2G47500	19491544	2	A/A	16.9	upstream_gene	P-loop nucleoside triphosphate hydrolases superfamily protein with CH (Calponin Homology) domain-containing protein
AT2G47550	19511346	2	C/C	15.091	synonymous	Plant invertase/pectin methylesterase inhibitor superfamily
AT2G47600	19531942	2	C/C	14.066	upstream_gene	magnesium/proton exchanger
AT2G47670	19547015	2	A/A	15.957	upstream_gene	Plant invertase/pectin methylesterase inhibitor superfamily protein
AT2G47690	19550389	2	G/G	14.843	upstream_gene	NADH-ubiquinone oxidoreductase-like protein
AT2G47740	19564206	2	A/A	16.048	upstream_gene	tRNA-Gly
AT2G48020	19649184	2	C/C	14.178	downstream_gene	Major facilitator superfamily protein
